# Multi-trait and multi-environment genomic prediction enhances yield components improvement in durum wheat

**DOI:** 10.3389/fpls.2026.1759897

**Published:** 2026-02-23

**Authors:** Damiano Puglisi, José Crossa, Jaime Cuevas, Fabio Fania, Sanaz Afshari-Behbahanizadeh, Paolo Vitale, Pasquale De Vita

**Affiliations:** 1 CREA – Research Centre for Cereal and Industrial Crops (CREA-CI) Consiglio per la Ricerca in Agricoltura e l’Analisi dell’Economia Agraria, Foggia, Italy; 2 Colegio de Postgraduados, Texcoco, Estado de México, Mexico; 3 Division de Ciencias, Ingeniería y Tecnología (DCIT), Universidad Autónoma del Estado de Quintana Roo, Chetumal, Quintana Roo, Mexico; 4 Department of Agriculture, Food, Natural Science, Engineering (DAFNE), University of Foggia, Foggia, Italy; 5 International Maize and Wheat Improvement Center (CIMMYT), Texcoco, Estado de México, Mexico

**Keywords:** gene-based relationship matrix, genomic selection, GxE interaction, SNP markers, sowing time, yield-related traits

## Abstract

Durum wheat [*Triticum turgidum* L. ssp. *durum* (Desf.) Husn.] is a staple crop for the pasta and semolina industries, particularly in Mediterranean and semi-arid regions where climate variability poses major challenges to yield stability. This study evaluates the performance of single-environment (SE), multi-trait (MT), multi-environment (ME), and multi-trait–multi-environment (MTME) genomic prediction models across seven key traits, such as grain number per spike, grain weight per spike, number of spikelets per spike, spike length, spike weight, heading date, and plant height. Using genomic (G) and target gene-based (G2) relationship matrices with two cross-validation scenarios (CV1 and CV2), MTME models achieved the highest prediction accuracies, particularly under CV2 and sowing-by-season grouping. Modeling G2 information improved predictions for morpho-phenological traits (i.e. heading date and plant height), confirming the utility of functional allele data for capturing gene effects. MTME models effectively leveraged inter-trait and inter-environment covariance, providing biologically realistic predictions of genotype performance across simulated Mediterranean environments. These findings establish MTME genomic prediction as a powerful and scalable framework for climate-resilient durum wheat improvement, supporting predictive and data-driven breeding pipelines aimed at enhancing genetic gain and stability across years and environments.

## Introduction

Durum wheat [*Triticum turgidum* L. ssp. *durum* (Desf.) Husn.] is a cereal crop of significant global economic and nutritional importance, providing raw material for pasta, couscous, bulgur, and other semolina-based foods (Hammami and Sissons, 2020). It occupies approximately 13 million hectares worldwide and produces between 30–35 million metric tons annually (Martínez-Moreno et al., 2022). The Mediterranean Basin remains the heart of its cultivation, encompassing southern European countries such as Italy, Spain, Greece, and France, as well as North African nations including Morocco, Tunisia, and Algeria. Beyond this region, durum production has expanded into the Americas (Mexico, Argentina, Chile, Canada, and the United States) to satisfy growing demand for premium semolina and pasta (De Vita and Taranto, 2019). Due to its resilience to heat and drought, durum wheat is considered a strategic crop for ensuring food security and climate-change adaptation.

Despite remarkable genetic progress, breeding for durum wheat adaptation to the increasingly erratic Mediterranean climate remains a formidable challenge. Irregular rainfall, recurrent droughts, and temperature extremes generate strong genotype × environment (G×E) and/or genotype × year (G×Y) interactions, which mask true genetic values and hinder selection efficiency, making it harder to identify best-performing genotypes (Lopez-Cruz et al., 2015). Traditional phenotypic selection, though historically successful, relies on multi-environment and multi-year field evaluations and large experimental networks, making it slow and resource-consuming. Moreover, correlations among environments are often weak, limiting the ability to extrapolate genotype performance to untested seasons or sites. These limitations underscore the need for predictive approaches that leverage genetic correlations across traits and environments to accurately estimate the genetic values of candidate lines.

The advent of genomic selection (GS) revolutionized quantitative breeding by allowing estimation of total genetic value from dense genome-wide markers (Meuwissen et al., 2001; Crossa et al., 2017). Through genomic prediction (GP), breeders can select superior genotypes based on genomic estimated breeding values (GEBVs) before phenotyping, thereby shortening breeding cycles and improving accuracy, ultimately increasing genetic gain.

Multi-trait (MT) models capitalize on the principle of *borrowing strength* from correlated traits. As a result, MT genomic prediction is becoming more common. By analyzing several traits together and leveraging their variance-covariance structure, it can boost target-trait prediction accuracy (Jia and Jannink, 2012). Numerous studies have shown that MT models improve prediction accuracy for low heritable target traits by borrowing information from correlated secondary traits with higher heritability (Crossa et al., 2017; Montesinos-López et al., 2018a, 2019a, 2022; Cuevas et al., 2023; Vitale et al., 2024).

Conversely, multi-environment (ME) models extend prediction across spatial and temporal dimensions by explicitly accounting for G×E and G×Y interactions. In Mediterranean durum wheat area, these interactions arise from gradients in rainfall, temperature, and soil fertility across locations and seasons. ME models accommodate this complexity through covariance structures or kernels that capture environmental similarity from climatic or phenotypic data (Jarquín et al., 2014; Lopez-Cruz et al., 2015; Cuevas et al., 2016, 2017; Montesinos-López et al., 2018a; Puglisi et al., 2021).

The integration of both concepts in multi-trait–multi-environment (MTME) models represents a significant methodological advance. MTME models jointly model traits and environmental covariance structures. Recent applications in wheat, maize, and rice have demonstrated the superiority of MTME frameworks for predicting untested genotypes across new environments (Montesinos-López et al., 2016, 2018b, 2018c, 2019a). Nevertheless, durum wheat remains underrepresented, particularly in the strong climatic gradients of the Mediterranean Basin, where its potential to improve predictive accuracy and biological interpretation has yet to be fully quantified.

In parallel with these modeling considerations, the representation of genomic information profoundly affects predictive performance. Most GS applications rely on a standard genomic relationship matrix (G) derived from SNP markers, which effectively captures additive genetic relationships but overlooks functional allelic effects. In recent years, several alternatives to the conventional G matrix have been proposed in the literature (Su et al., 2014; Lopes et al., 2017; Medina et al., 2021; Esposito et al., 2023). However, constructing G from a small set of well-characterized target genes remains largely unexplored.

This study aims to provide a comprehensive evaluation of multi-trait–multi-environment genomic prediction for durum wheat improvement under Mediterranean conditions. Specifically, we compared four genomic prediction frameworks: (i) single-environment and single trait (SE), (ii) single-environment and multi-trait (MT), (iii) multi-environment single-trait (ME), and (iv) multi-trait and multi-environment (MTME). Each model is evaluated under two cross-validation schemes (CV1 and CV2) and with two relationship matrices: G, built from genome-wide markers, and G2, built from specific target genes.

This analysis focused on seven key traits, such as grain number per spike (GN), grain weight per spike (GW), number of spikelets per spike (NS), spike length (SL), spike weight (SW), heading date (HD), and plant height (PH), which jointly define productivity, developmental timing, and resilience under Mediterranean field conditions. These traits provide an integrated biological basis for testing how trait interdependence and environmental variability can be exploited to enhance prediction accuracy.

By benchmarking these models across traits, sowing dates, and multiple years, this work offered an extensive assessment to date of MTME genomic prediction in durum wheat. The results identify optimal modeling strategies and data representations that maximize predictive accuracy, offering a data-driven predictive framework for next-generation, climate-resilient breeding pipelines.

## Materials, methods, and models

### Plant material and phenotyping

A panel of 186 durum wheat genotypes was used for this study (**Supplementary Table 1**). This panel has already been employed in a previous study published by Puglisi et al. (2025) to implement genomic prediction models for morpho-phenological traits [plant height (PH, cm); heading date (HD) and flowering time (FT), expressed as growing degree days (gdd)]. The same study also describes the genotyping procedures used for this panel, providing a comprehensive genomic characterization of the material.

The genotypes were grown across a few simulated Mediterranean conditions, including three sowing dates (early, optimal, and delayed) over three consecutive growing seasons (from 2021–22 to 2023-24) at the CREA Research Centre for Cereal and Industrial Crops, Foggia, Italy (41° 51′ N, 15° 52′ E, 60 m elevation).

In addition to morpho-phenological traits (i.e., HD, FT, and PH) previously reported (Puglisi et al., 2025), at the ripening stage (Zadoks stage 90) (Zadoks et al., 1974), the entire panel was manually harvested to evaluate yield-related traits for each sowing-by-season combination. The grain number per spike (GN), grain weight per spike (GW), number of spikelets per spike (NS), spike length (SL), and spike weight (SW) were measured in 25 randomly selected spikes from each plot across all sowing dates and growing seasons.

### Adjusted means

Adjusted means (Best linear unbiased estimates, BLUEs) were computed from raw measurements using the following linear mixed model:

(1) $${\text{y}}_{\text{sjr}}\;=\text{ µ }+{\text{Gen}}_{\text{s}}+{\text{ Rep}}_{\text{j}}+{\text{Block}}_{\text{r}}({\text{Rep}}_{\text{j}})+{\text{ϵ}}_{\text{sjr}}$$

where ${\text{y}}_{\text{sjr}}$ is the response variable, µ is the general mean, ${\text{Gen}}_{s}$ is the fixed effect of the $s^{th}$ genotype and follow a normal distribution with mean 0 and variance equals to $\sigma_{g}^{2}$, that is ${\text{Gen}}_{s}∼NIID(0,\sigma_{g}^{2})$, the ${\text{Rep}}_{\text{j}}$ is effect for the $j^{th}$ replication, $Block_{r}(Rep_{j})$ is the random interaction effect of $r^{th}$ block within the $j^{th}$ replication, finally ${\text{ϵ}}_{sjr}$ is the residual terms normally distributed with mean 0 and variance equals to $\sigma_{\text{ϵ}}^{2}$, that is ${\text{ϵ}}_{sjr}\;∼NIID(0,\sigma_{\text{ϵ}}^{2})$. This model (Equation 1) was implemented using the lme4 package (Bates et al., 2015) in R 4.0.3 statistical environment (R Core Team, 2020).

### Correlations and genotype-by-environment analysis

Pairwise correlations among traits were calculated for each environment and visualized using two complementary approaches in R 4.0.3 statistical environment (R Core Team, 2020): i) relationships among traits across all environments were explored using the GGally package (Schloerke et al., 2025) via the *ggpairs* function; ii) for each trait, correlations between environments were displayed as heatmaps using a custom ggplot2-based function (Wickham, 2016). The function reordered rows and columns based on hierarchical clustering.

Site regression (SREG) analysis was used to obtain more information on genotype-by-environment (GxE) interactions. SREG analysis was performed according to Samonte et al. (2005) using imputed values in GEA-R version 4.1 (Samonte et al., 2005; Pacheco et al., 2018), and scores of genotypes were plotted on a biplot.

### Statistical genomic prediction models

Genomic Best Linear Unbiased Prediction (GBLUP) is one of the foundational models in modern genomic selection. It extends the classical Best Linear Unbiased Prediction (BLUP) framework by replacing the pedigree-based additive relationship matrix (A) with a genomic relationship matrix (G) derived from molecular marker data. This allows breeders to predict the genetic merit of unphenotyped individuals using genotypic information from dense genome-wide markers. GBLUP assumes that all marker effects contribute equally and independently to the total genetic variance, and it provides Best Linear Unbiased Predictions (BLUPs) of genomic breeding values (GEBVs).

#### Single-trait and single-environment (SE)

The basic linear mixed model used in genomic prediction can be expressed as:

(2) y = Xb + Zu + ϵ

where y  is the vector of the BLUEs, X is the incidence matrix for fixed effects (e.g., intercept, environment), b is the vector of fixed effects, Z is the incidence matrix relating observations to random genetic effects, u is the vector of random genomic breeding values (GEBVs), and ϵ is the vector of random residuals. (Equation 2)

In GBLUP, the covariance structure of the genomic effects is defined by the genomic relationship matrix (G), which measures the realized genetic similarity among individuals. The matrix G is calculated using standardized marker genotypes as follows:

(3) G=ZZ'2 Σ pi (1 − pi)

where Z is the centered and scaled genotype matrix (values coded as 0, 1, or 2 for homozygous, heterozygous, and alternate homozygous alleles), and $p_{i}$ represents the allele frequency at the *i*^th^ marker. This formulation (Equation 3), proposed by VanRaden (2008), ensures that G approximates the expected additive genetic relationship among individuals.

#### Multi-trait and single-environment (MT)

The multi-trait approach was carried out using the following model:

(4) Y=1μ'+U+E

where Y is the matrix of the BLUEs for each trait and individual, μ is the vector of the intercepts for each trait $\text{μ}=({\text{μ}}_{1}\ldots {\text{μ}}_{n})$ where n is the number of traits, U is the matrix of random effects, and E is the matrix of the residuals (Equation 4). The residuals are assumed to be independent and identically distributed (IID), following a multivariate normal (MVN) distribution with zero mean and covariance matrix. To explore the model’s details, see Pérez-Rodríguez and de los Campos (2022) (Pérez-Rodríguez and de los Campos, 2022).

#### Single-trait and multi-environment (ME)

We modeled G×E interaction within the genomic prediction model as follows:

(5) $$y_{ij}=\mu +E_{i}+L_{j}+{ɡ}_{j}+E{ɡ}_{ij}+\epsilon_{ij}$$

where $y_{ij}$ is the BLUE value for each *j*^th^ line in each *i*^th^ environment, μ is the intercept, $E_{i}$ is the effect of the *i*^th^ environment, $L_{j}$ is the effect of the of the *j*^th^ line, ɡ = ( ${ɡ}_{1}\ldots {ɡ}_{j})$ is the vector of the genetic effects, which follows a normal distribution $ɡ\;∼\;N\left(0,\left({\text{Z}}_{\text{g}}{\text{GZ'}}_{\text{g}}\right)\sigma_{ɡ}^{2}\right)$, with ${\text{Z}}_{\text{g}}$ referring to the incidence matrix, and $\sigma_{g}^{2}$ the variance component associated with ɡ. We also assumed that $Eɡ=\left\{E{ɡ}_{ij}\right\}\;∼N\left(0,\left({\text{Z}}_{\text{g}}{\text{GZ'}}_{\text{g}}\right)\#\left({\text{Z}}_{\text{E}}{\text{Z'}}_{\text{E}}\right)\sigma_{Eɡ}^{2}\right)$ is the element-wise product, ${\text{Z}}_{\text{E}}$ refers to the incidence matrix for the environments, and $\sigma_{Eɡ}^{2}$ is the variance term for the G×E interaction. Finally, $\epsilon_{ij}$ corresponds to the residuals with mean 0 and variance $\sigma_{\text{ϵ}}^{2}$ (Equation 5).

#### Multi-trait and multi-environment (MTME)

The multi-trait and multi-environment approach can be summarized as follows:

(6) $$\text{Y}=\text{Xβ}+{\text{Z}}_{1}{\text{b}}_{1}+{\text{Z}}_{2}{\text{b}}_{2}+\text{E}$$

where Y is the input of BLUEs for each trait and for each environment, X is the design matrix for the environmental fixed effects, β is the matrix of the beta coefficients, ${\text{Z}}_{1}$ is the incidence matrix for the genotypes, ${\text{b}}_{1}$is the matrix of the random effects for the genotypes, ${\text{Z}}_{2}$ is the incidence matrix for the G×E interaction, ${\text{b}}_{2}$ is the matrix of the random effects for the G×E interaction, and E is the matrix of the residuals. We assumed that ${\text{b}}_{1}$ was distributed under a matrix-variate normal distribution as ${\text{b}}_{1}\;∼MVN(0,\text{G},{\text{Σ}}_{t})$ where ${\text{Σ}}_{t}$ is the unstructured variance-covariance matrix across traits. Additionally, ${\text{b}}_{2}\;∼MVN(0,{\text{Σ}}_{E}⊗\text{G},{\text{Σ}}_{t})$ where ${\text{Σ}}_{E}$ is the unstructured variance-covariance matrix across environments and ⊗ is the Kronecker product (Equation 6). Additional information for this model was shown by Montesinos-López et al. (2016, 2019a) (Montesinos-López et al., 2016, 2019a).

Finally, G was computed using genome wide Single-Nucleotide Polymorphisms (SNPs) markers. Furthermore, a complementary allelic matrix ( G2), constructed from the allelic combinations of specific target genes such as *Vrn-A1*, *Ppd-A1*, *Ppd-B1*, and *Rht-B1* (Puglisi et al., 2025), was incorporated into the basic linear mixed model across all GP models implemented (i.e., SE, ME, MT, and MTME).

GP models were performed in the R environment using the package BGLR (Pérez and de los Campos, 2014). A high-performing computer has been used to carry out all the analyses. This was characterized by four nodes, each of which included 96 cores and 500GB of RAM.

### Fundamentals and interpretation of cross-validation schemes

Cross-validation (CV) is a conventional method for assessing predictive performance in genomic prediction studies. In genomic prediction, CV1 and CV2 refer to two common CV strategies used to evaluate genomic model performance across distinct prediction scenarios. These schemes simulate realistic breeding conditions and make predictions for either new genotypes or new environments. Here, we conducted 10 cycles of 5-fold random cross-validation to assess model predictability under SE, MT, ME, and MTME. The strategies MT, ME, and MTME were performed using both CV1 and CV2.

The CV for SE was performed within each year-sowing date combination separately. This consisted of randomly partitioning the population into five sets, with four used to train the model and the remaining set used to test it. This process is usually performed until all sets have been used for testing at least once. Then, this process was repeated for 10 cycles.

Additionally, both CV1 and CV2 are well explained by Lado et al. (2018) and Jarquín et al. (2014) for multi-trait and multi-environment, respectively. However, we provided brief descriptions of both cross-validation methods in the next paragraphs.

*CV1 – Prediction of Unseen Genotypes (within known environments and traits).*

The goal of CV1 is to assess how well the model predicts new lines (genotypes) that have never been phenotyped but are genotyped, assuming that the environments and/or traits are already represented in the training data. In CV1, the dataset is divided by genotypes: some are used for training and others (never seen during training) for testing. All environments are represented in both subsets. This setup mimics a *“new candidate line prediction”* scenario, where breeders aim to predict the performance of untested genotypes before field evaluation. CV1 primarily tests the model’s ability to generalize across genotypes, relying on molecular marker information and genetic relationships. Prediction accuracy in CV1 reflects how effectively genomic data alone can capture genetic value when environmental effects are already known.

*CV2 – Prediction of tested genotypes (within known environments and traits).*

The goal of CV2 is to evaluate how well a model predicts the performance of known genotypes in new environments (evaluated for other genotypes) for the primary (target) trait. In this setting, target genotypes are observed during training in some environments and/or for both primary and secondary traits, while their phenotypic records for the primary trait are missing in the target environment. Information from other genotypes evaluated in the target environment, as well as from secondary traits measured on the target genotypes, is available and used for model training. In CV2, the dataset is divided by environments and/or traits rather than by genotypes. The same set of genotypes is included in both training and testing, but phenotypic records from some environments are omitted during training. In this cross-validation strategy, the aim is to predict the performance of known genotypes in environments and/or traits for which they have no observed phenotypes.

## Results

### Phenotypic distribution of the yield-related traits

**Figure 1:**
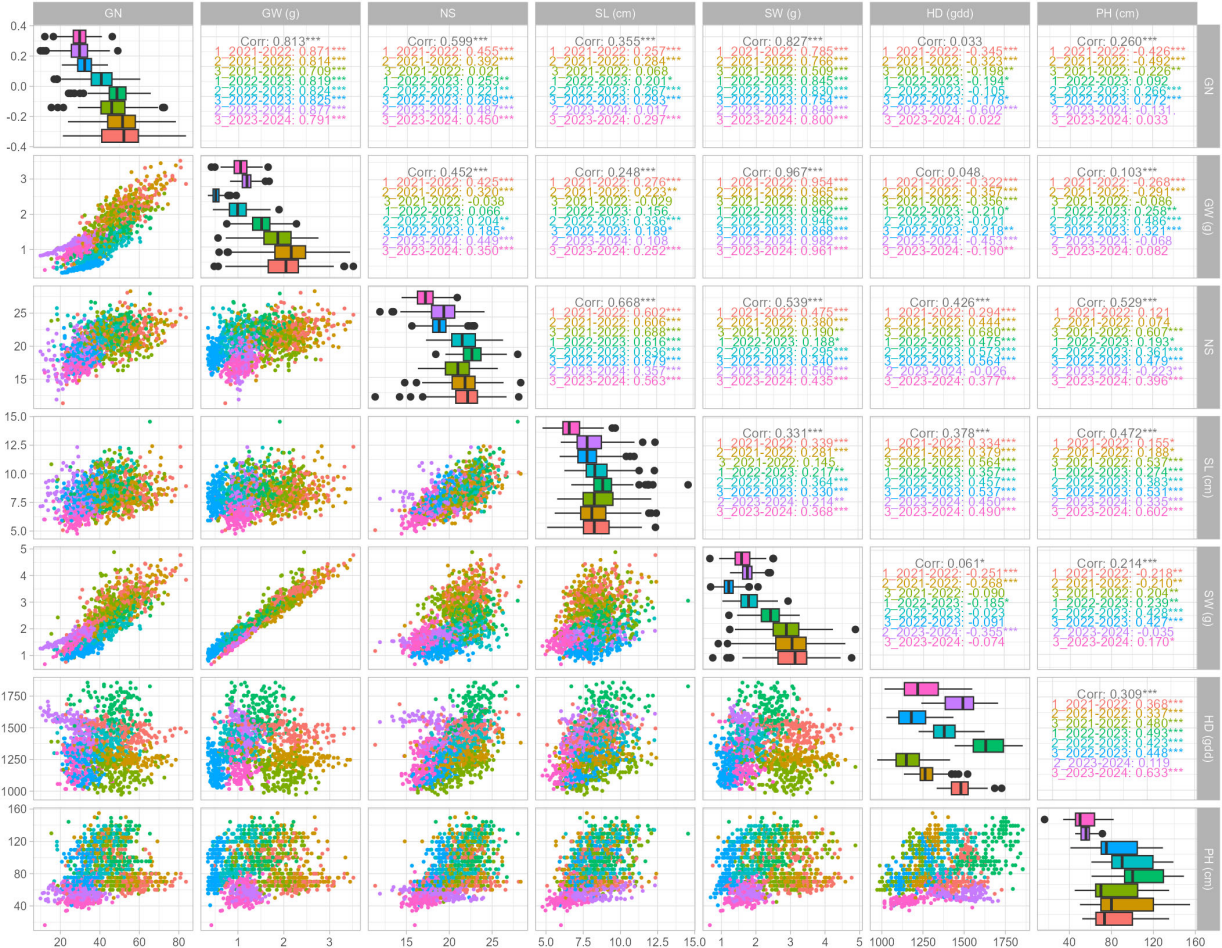
Pairwise Pearson’s correlations were conducted using the adjusted means of yield component traits and morpho-phenological traits. Three distinct sowing dates: early, optimal, and delayed, are indicated by different colors, and are displayed for each growing season, except for the 2023–2024 season, which has only two sowing dates. The asterisks indicate the significance of the correlation (***<0.001, **<0.01, *<0.05). The lower graph displays the pairwise scatter correlation plots, while the diagonal plots show the phenotypic data distribution.

Adjusted means (BLUEs) of the phenotypic data collected over three consecutive growing seasons (2021–2024) and multiple sowing-by-season combinations (environments) reveal substantial variability across traits and conditions (see diagonal plots of **Figure 1**; **Supplementary Table 2**).

The phenotypic distribution of HD and PH were strongly influenced by sowing date and growing season, respectively, as previously reported (Puglisi et al., 2025). Similarly, a wide range of variation was observed across environments for yield-related traits, indicating the influence of both genetic and environmental factors.

GN showed the widest phenotypic range, with values ranging from 9.89 to 83.65 grains, and the coefficient of variation ranging between 0.154 and 0.251. The broad-sense heritability (h²) across environments varied from 0.352 to 0.553, suggesting moderate genetic control.

GW ranged from 0.343 to 3.518 g, with coefficient of variation ranging from 0.132 to 0.282. The heritability values were moderate (h² = 0.302–0.533), reflecting a stable genetic influence under different environmental conditions.

NS ranged from 11.35 to 28.33, showing relatively low coefficient of variations (0.070–0.113), suggesting more stable expression across environments. Heritability values were consistently high, up to 0.642, pointing to a stronger genetic determinism.

SL values were between 4.765 and 14.555 cm, with coefficient of variation ranging from 0.127 to 0.162 and high heritability (h² = 0.674–0.736) across all evaluated environments, indicating that this trait is under strong genetic control.

SW showed a wide range (0.669 to 4.880 g) and moderate coefficient of variation (0.128–0.236). Heritability values ranged from 0.355 to 0.540, again reflecting moderate genetic contribution.

In general, traits such as SL and NS exhibited higher heritability, suggesting they are less affected by environmental variation and may respond better to selection. Conversely, GN and GW, while showing significant phenotypic variability, presented slightly lower heritability estimates, likely due to stronger genotype-by-environment interactions.

### Correlation analysis and genotype × environment interactions

#### Pairwise Pearson’s correlations

Pairwise Pearson’s correlations showed moderate to high associations among yield-related traits (i.e., GN, GW, SW, NS, and SL), while morpho-phenological traits (i.e., HD and PH) were only weakly correlated with yield components (**Figure 1**). The strongest relationships were detected between SW and GW (r = 0.97) and between SW and GN (r = 0.83), confirming that SW is a composite measure integrating both kernel number and grain mass. Correlation patterns across sowing-by-season combinations were consistent (**Figure 1**), indicating that these relationships are stable across environmental gradients. **Supplementary Figure 1** illustrates the pairwise correlations between environments, confirming the stability of trait–trait relationships across sowing-by-season combinations.

#### Site regression model

**Figure 2:**
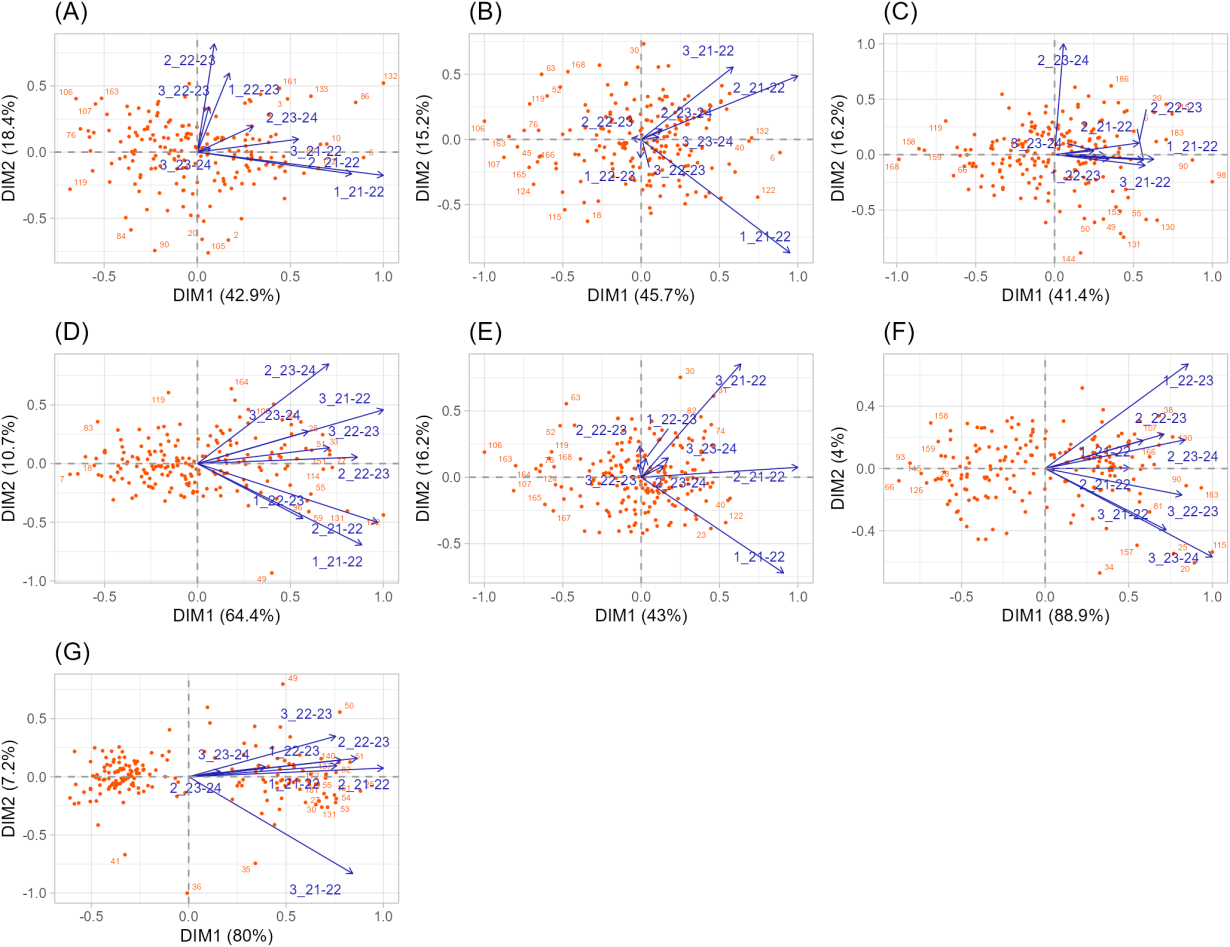
Genotype plus Genotype x Environment (G+GE) biplot obtained from sites regression (SREG) analysis across the eight sowing-by-season combination for each yield-related and morpho-phenological traits: **(A)** Grain Number per spike (GN), **(B)** Grain Weight per spike (GW), **(C)** Number of Spikelets per spike (NS), **(D)** Spike Length (SL), **(E)** Spike Weight (SW), **(F)** Heading Date (HD), and **(G)** Plant Height (PH). Each GGE Biplot shows components 1 and 2 of the total variation G+GE.

To further dissect the G×E structure, a site regression (SREG) model (Crossa et al., 2002) was fitted using imputed phenotypic data, and genotype plus genotype × environment (G+GE) biplots were constructed for each trait (**Figures 2A–G**). The first two principal components explained a large proportion of the total G+GE variation, ranging from 57.6% (NS) to 75.1% (SL) for yield components and exceeding 87% for morpho-phenological traits (HD and PH). This indicates that a two-dimensional biplot effectively captured the main patterns of genotypic adaptation and environmental differentiation.

For GN, the environments corresponding to the 2022–2023 cycle formed a tightly associated cluster (r = 0.64***), clearly separated from those of 2021–2022 and 2023–2024 (**Figure 2A**). This suggests that the 2022–2023 season imposed specific environmental conditions, likely a combination of favorable temperature and rainfall, that consistently influenced spike fertility and grain set. GW and SW exhibited similar biplot structures (**Figures 2B, E**), reinforcing the shared physiological basis of sink–source dynamics. In both cases, environments from 2022–2023 clustered together, while 2021–2022 and 2023–2024 formed distinct groups, indicating strong year-specific effects. Genotypes G030 and G031 were identified as the best-performing in the late sowing date of 2021–2022 growing season (3_2021-2022), while G167 and G181 showed the lowest performance, exemplifying specific adaptation to environmental niches.

The NS presented a distinct pattern (**Figure 2C**): most environments were moderately correlated (r ≈ 0.5), but the optimal sowing date of 2023–2024 growing season (2_2023-2024) behaved as an outlier with an opposite response. This deviation likely reflects contrasting environmental conditions (e.g., water deficit, high temperature) during spikelet differentiation, emphasizing the sensitivity of this trait to early developmental stress. For SL, environments were generally correlated but arranged in a fan-shaped configuration (**Figure 2D**), suggesting gradual environmental differentiation typical of traits influenced by continuous climatic gradients rather than discrete stress factors.

Finally, morpho-phenological traits showed high stability across environments (**Figures 2F, G**). Both HD and PH formed compact clusters with strong positive correlations among most sowing-by-season combinations. Only environment 3_2021–2022 for HD and 1_2022–2023 for PH slightly deviated from the general pattern, contributing modestly to the total G×E variance. The very high proportion of variation explained by the first two factors (87.3% for HD, 92.9% for PH) confirms that these traits are primarily under genetic control and exhibit limited environmental sensitivity. **Supplementary Figure 1** further visualizes these patterns, confirming that morpho-phenological traits are stable across sowing years and conditions.

#### From correlations and G×E interactions to genomic prediction frameworks

The combined correlation and SREG analyses, as they provide a clear picture of trait interdependence and environmental structuring in Mediterranean durum wheat, establish the phenotypic foundation for the GP results that follow.

The consistent clustering of environments by year and sowing condition highlights structured environmental variability, while the strong associations among yield components reveal physiological linkages exploitable by MT GP models. Together, these findings confirm that both inter-trait and inter-environment correlations are key determinants of durum wheat performance.

### Predictive ability of GP models for yield-related traits

#### Grain number per spike

**Figure 3:**
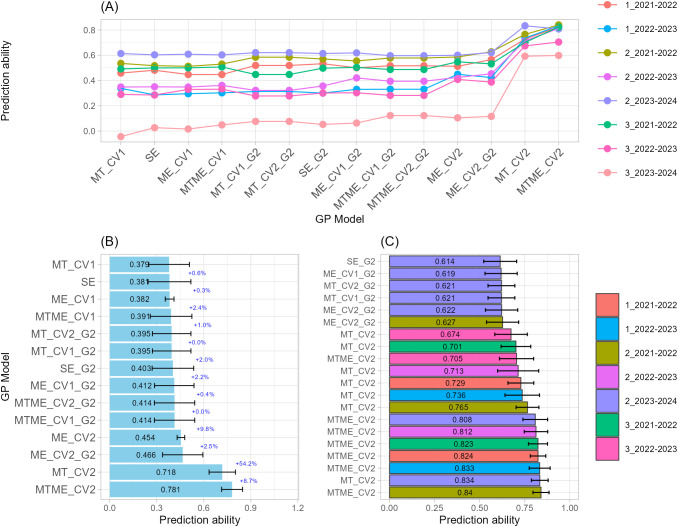
Single-trait-single-environment (SE), multi-trait-single-environment (MT), single-trait-multi-environment (ME), and multi-trait–multi-environment (MTME) genomic prediction (GP) models used to predict Grain Number per spike (GN) using both genomic (G) and allelic (G2) relationship matrices with two cross-validation schemes (CV1 and CV2). **(A)** Overall prediction ability for each sowing-by-season (environment) combination and GP models used; **(B)** Prediction ability across sowing-by-season (environment) combination for each GP models used. Error bars indicate variability across environments, while percentage values indicate the improvement relative to the model immediately above; **(C)** Prediction ability of top-20 GP models used.

The prediction of GN revealed consistent gains in predictive ability when multi-trait and multi-environment genomic prediction models were used, even in environment 3_2023-2024, where prediction accuracy was particularly low across all GP models (**Figure 3A**). This reduced performance is likely associated with the stressful conditions observed during the late sowing date of 2023–2024 growing season, characterized by higher temperatures and lower precipitation (Puglisi et al., 2025), which may have intensified crop stress and increased environmental variability, thereby limiting the predictive ability of the GP models. Across sowing-by-season combination (environment), models integrating multiple traits (MT) and both traits and environments (MTME) implemented with CV2 and purely genomic (G) approaches, achieved the highest overall correlations of 0.71 and 0.78, respectively (**Figure 3B**). Across the Top-20 model, prediction ability ranged from 0.84 to 0.61, with standard deviations from 0.04 to 0.11, suggesting moderate variability across CV partitions (**Figure 3C**; **Supplementary Table 3**). Across the growing seasons, the optimal sowing date appeared most frequently among the best-performing combinations, indicating high reproducibility for those specific sowing-by-season conditions.

Within the MTME framework, the MTME_CV2 models dominated the top of the ranking, occupying nearly half of the Top-20 cases. The best-performing case achieved r = 0.8402 (MTME_CV2, 2_2021–2022), followed closely by MT_CV2 at r = 0.8335 (2_2023–2024). Other strong MTME_CV2 predictions were recorded for environments 1_2022–2023 and 3_2021–2022, with values above 0.82, confirming the robustness of multi-trait multi-environment models. MT also performed strongly, particularly using MT_CV2 in environments 2_2023–2024 and 2_2021–2022, with correlations between 0.76 and 0.83. In contrast, SE produced lower accuracies, indicating that models exploiting trait correlations or environmental structures substantially improved prediction. The inclusion of the G2 kernel, which accounts for allelic combinations of key genes, provided modest improvements over G alone in SE and ME structures. For instance, SE_G2 completed the Top-20 list, showing the lowest correlation (r = 0.6139) but still consistent across folds (SD = 0.0913). Anyway, GP models incorporating allelic information (G2) through ME_CV2_G2 or MT_CV2_G2 also appeared among the higher ranks, with r = 0.62–0.63 in environments 2_2021–2022 and 2_2023–2024. Although their accuracy was below MTME and MT models, they demonstrated additional stability across replicates.

#### Grain weight per spike

**Figure 4:**
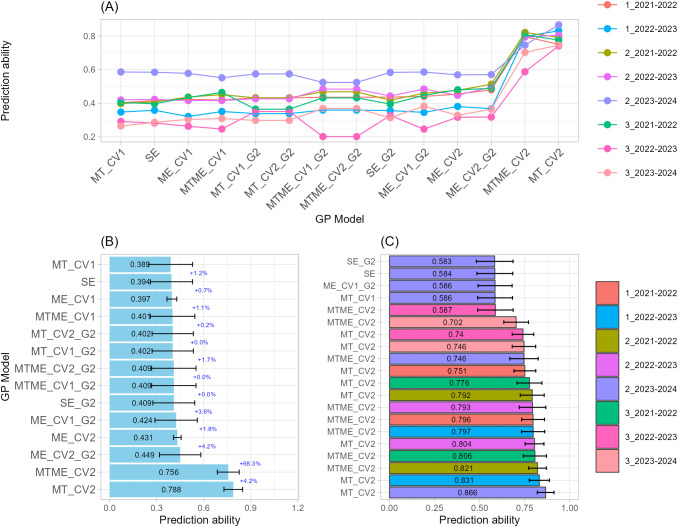
Single-trait-single-environment (SE), multi-trait-single-environment (MT), single-trait-multi-environment (ME), and multi-trait–multi-environment (MTME) genomic prediction (GP) models used to predict Grain Weight per spike (GW) using both genomic (G) and allelic (G2) relationship matrices with two cross-validation schemes (CV1 and CV2). **(A)** Overall prediction ability for each sowing-by-season (environment) combination and GP models used; **(B)** Prediction ability across sowing-by-season (environment) combination for each GP models used. Error bars indicate variability across environments, while percentage values indicate the improvement relative to the model immediately above; **(C)** Prediction ability of top-20 GP models used.

As previously observed for GN, the prediction of GW revealed that multi-trait and multi-environment genomic prediction models consistently achieved the highest levels of predictive ability (**Figure 4A**). Across sowing-by-season combination, models that integrate information across multiple traits (MT) or jointly across traits and environments (MTME) implemented with CV2 outperformed single-environment and purely genomic (G) approaches, achieved the highest overall correlations of 0.78 and 0.75, respectively (**Figure 4B**). Across the Top-20 model, prediction ability ranged from 0.84 to 0.61, with standard deviations less than 0.1, suggesting moderate variability across CV partitions (**Figure 4C**; **Supplementary Table 4**). In this case, within the MT framework, MT_CV2 models dominated the top of the ranking, with the best-performing exceeding 0.80 in three different sowing-by-season combinations in both early and optimal sowing date, indicating consistent benefits from integrating trait-level covariation even when the explicit environment structure is omitted. MTME_CV2 also showed strong performance, particularly in environments 2_2021–2022 and 3_2021–2022. These results confirm that multi-trait and multi-environment structures capture the complex G×E interactions influencing both grain traits (GW as well GN) with high fidelity.

SE and genomic-only approaches (SE, G) generally achieved lower correlations, 0.39 on prediction average across all environments. The inclusion of the G2 kernel, such as SE_G2 and ME_CV1_G2, showed lower but still acceptable correlations (around 0.40 and 0.44, respectively), confirming a modest yet stable contribution of key-gene allelic data. The lowest accuracies within the Top-20 list were observed for SE and SE_G2 models (r ≈ 0.58), which nevertheless maintained reproducibility across folds (SD ≈ 0.10).

#### Number of spikelets per spike

**Figure 5:**
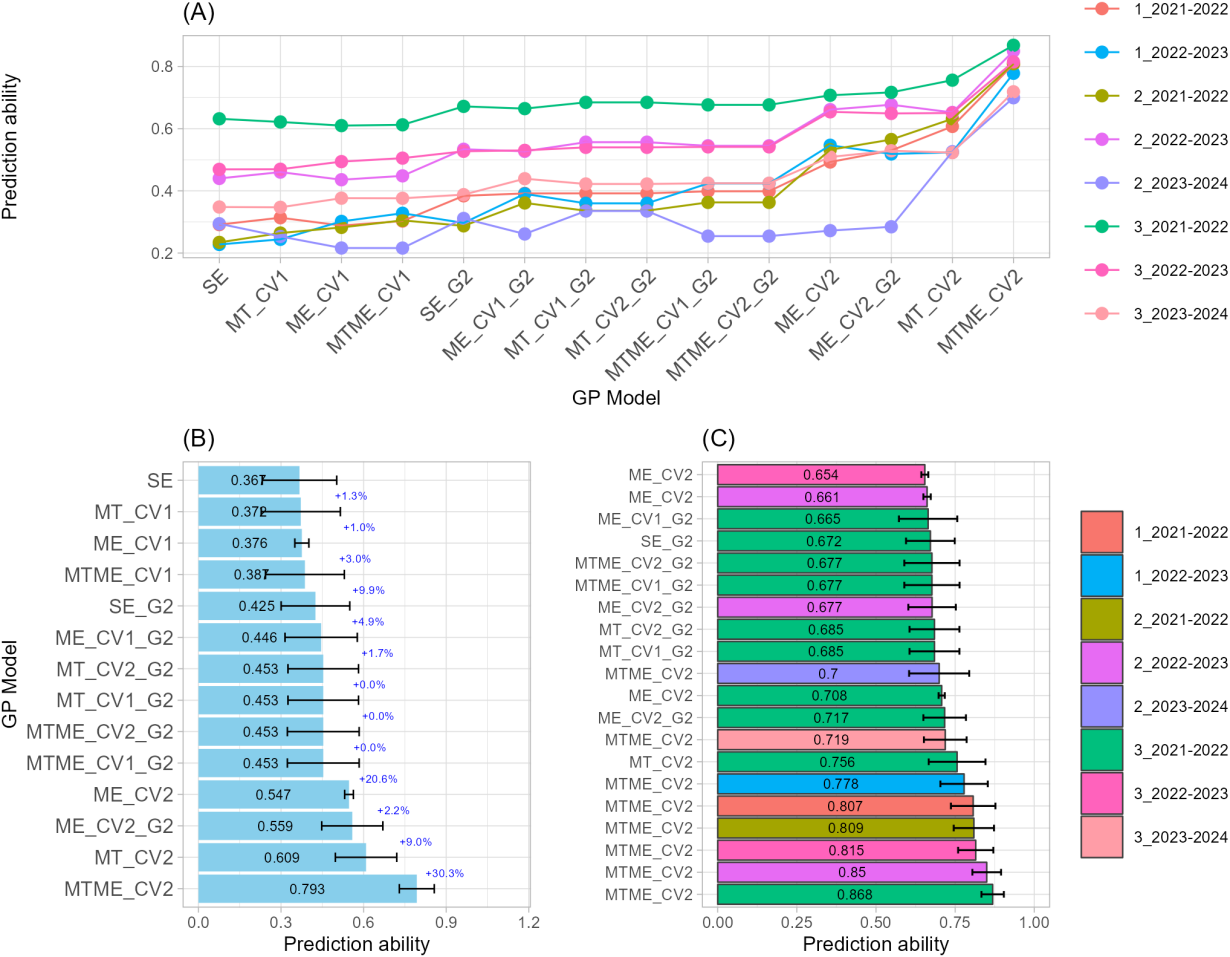
Single-trait-single-environment (SE), multi-trait-single-environment (MT), single-trait-multi-environment (ME), and multi-trait–multi-environment (MTME) genomic prediction (GP) models used to predict Number of Spikelets per spike (NS) using both genomic (G) and allelic (G2) relationship matrices with two cross-validation schemes (CV1 and CV2). **(A)** Overall prediction ability for each sowing-by-season (environment) combination and GP models used; **(B)** Prediction ability across sowing-by-season (environment) combination for each GP models used. Error bars indicate variability across environments, while percentage values indicate the improvement relative to the model immediately above; **(C)** Prediction ability of top-20 GP models used.

Regarding the NS, MTME_CV2 models consistently outperformed other genomic prediction structures with a prediction ability of 0.79 on average across all sowing-by-season combinations, indicating strong capacity to capture both genetic and environmental interactions (**Figures 5A, B**). For example, among the Top-20 models, MTME_CV2 ranked within the top six models (**Figure 5C**; **Supplementary Table 5**). The best performing model reached r = 0.8685 (MTME_CV2, 3_2021–2022), followed by MTME_CV2 models applied to environments 2_2022–2023 and 3_2022–2023 (r = 0.8498 and 0.8150, respectively). These results confirmed that MTME models were able to capture complex G×E structures underlying spikelet development more effectively than simpler models.

Intermediate accuracy was observed for MT_CV2 models (0.60 on average), indicating that trait-level integration contributed significantly even to the absence of explicit environmental modeling. ME and SE models incorporating the G2 kernel (SE_G2 and ME_CV2_G2) showed moderate predictive abilities, demonstrating that including allelic combinations of key genes improved prediction slightly over G-only structures (SE and ME_CV2). For instance, ME_CV2 and ME_CV2_G2 models also appeared among the top-performing cases, with accuracies between 0.70 and 0.71, providing evidence that adding allelic information (G2) further stabilized the models across folds. The lowest performing but still consistent models were SE_G2 and ME_CV1_G2 structures (r ≈ 0.66). Overall, models with G2 matrices showed marginal gains but did not reach the predictive strength of MTME configurations. Finally, the overall stability across folds and environments further supports the robustness of the MTME_CV2 framework as the optimal structure for NS prediction.

#### Spike length

**Figure 6:**
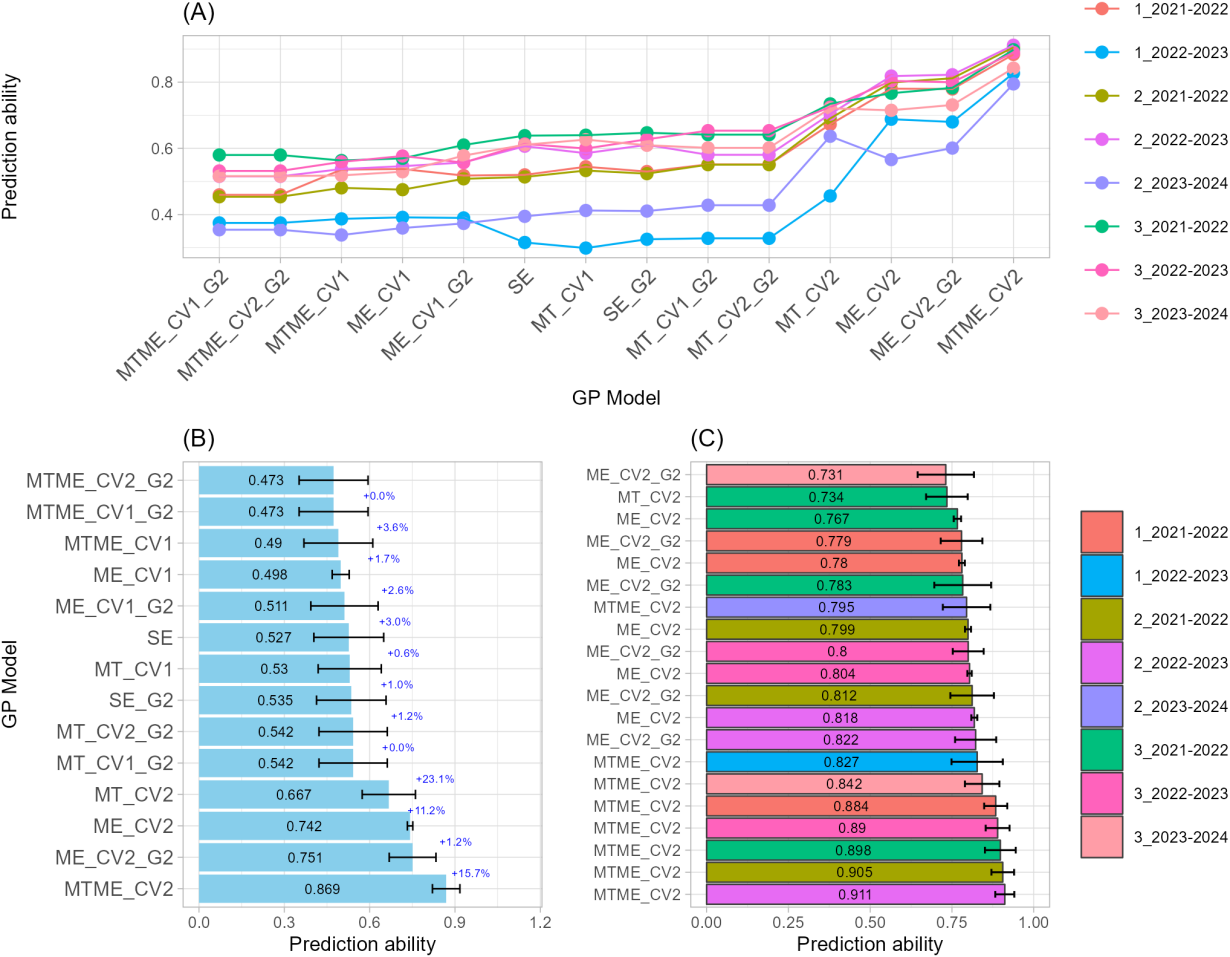
Single-trait-single-environment (SE), multi-trait-single-environment (MT), single-trait-multi-environment (ME), and multi-trait–multi-environment (MTME) genomic prediction (GP) models used to predict Spike Length (SL) using both genomic (G) and allelic (G2) relationship matrices with two cross-validation schemes (CV1 and CV2). **(A)** Overall prediction ability for each sowing-by-season (environment) combination and GP models used; **(B)** Prediction ability across sowing-by-season (environment) combination for each GP models used. Error bars indicate variability across environments, while percentage values indicate the improvement relative to the model immediately above; **(C)** Prediction ability of top-20 GP models used.

The prediction of SL showed very high predictive abilities for models incorporating both multi-trait and multi-environment information (**Figure 6A**). Across all sowing-by-season combinations, MTME_CV2 models produced the highest accuracies, with predictive correlations of 0.86 on average (**Figure 6B**). The top-performing MTME_CV2 model achieved r = 0.9053 for environment 2_2021–2022, followed closely by other MTME_CV2 models in environments 3_2021–2022 and 3_2022–2023 (r = 0.89–0.90) (**Figure 6C**; **Supplementary Table 6**). These results demonstrate a strong capacity of MTME models to account for environmental variability in SL prediction.

Multi-environment models, both with and without G2 kernels (ME_CV2 and ME_CV2_G2), achieved slightly lower but still high accuracies (r = 0.75). The addition of allelic information (G2) modestly enhanced prediction stability, particularly in environments 2_2022–2023, confirming its stabilizing effect under multi-environment conditions and reinforcing the importance of modeling allelic effects alongside environmental and trait correlations. By contrast, single-environment models or genomic-only structures were not among the top-performing configurations, as they were unable to capture the full extent of genotype × environment variation influencing SL. Across the Top-20 models, predictive ability ranged from 0.91 to 0.73, with a standard deviation ranging from 0.03 to 0.09, indicating high reproducibility of results across validation folds. Overall stability and high predictive ability confirm MTME_CV2 as the optimal structure for modeling SL in multi-year wheat trials.

#### Spike weight

**Figure 7:**
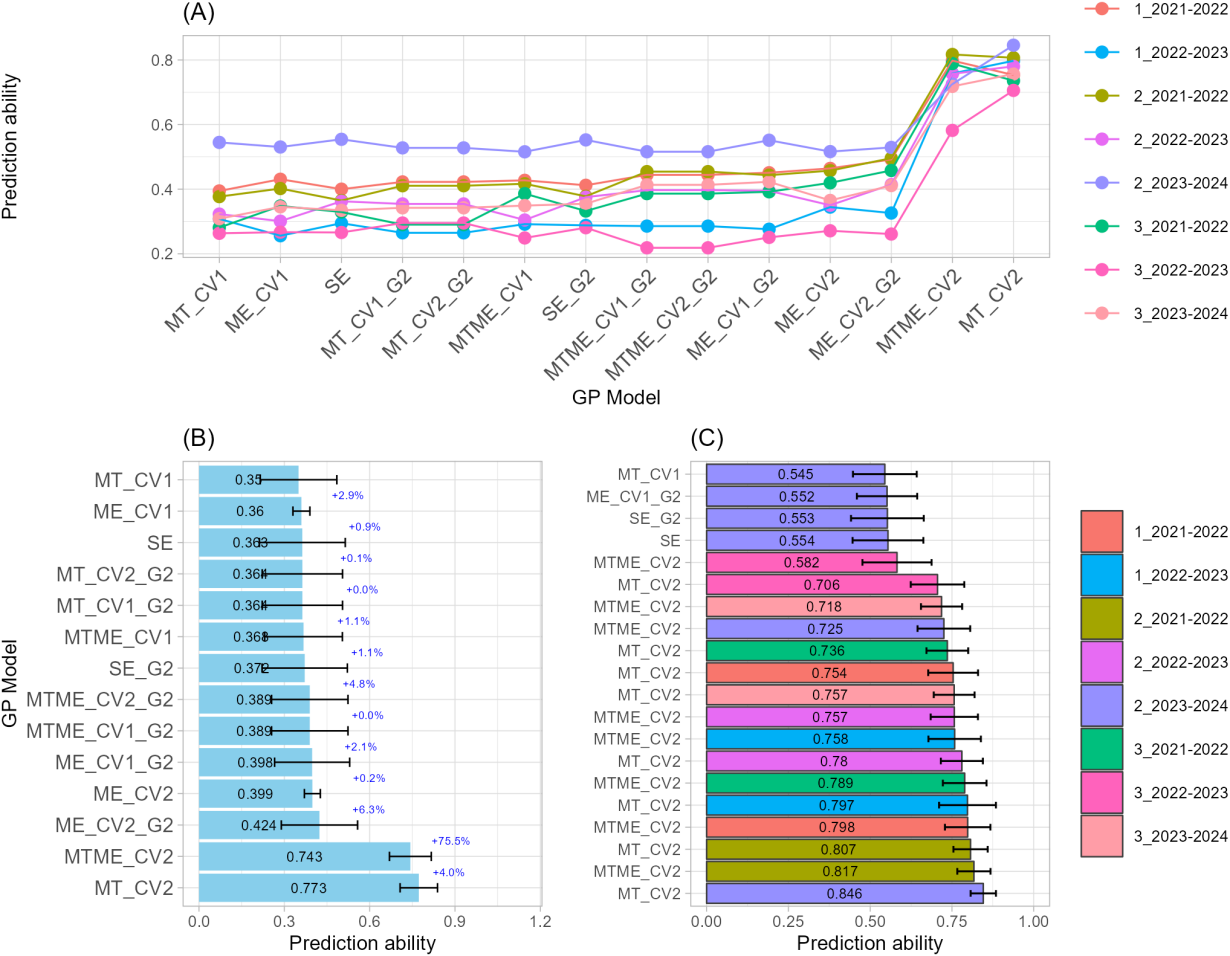
Single-trait-single-environment (SE), multi-trait-single-environment (MT), single-trait-multi-environment (ME), and multi-trait–multi-environment (MTME) genomic prediction (GP) models used to predict Spike Weight (SW) using both genomic (G) and allelic (G2) relationship matrices with two cross-validation schemes (CV1 and CV2). **(A)** Overall prediction ability for each sowing-by-season (environment) combination and GP models used. Error bars indicate variability across environments, while percentage values indicate the improvement relative to the model immediately above; **(B)** Prediction ability across sowing-by-season (environment) combination for each GP models used; **(C)** Prediction ability of top-20 GP models used.

As previously observed for both grain traits (GN and GW), the prediction of SW revealed consistent gains in predictive ability when multi-trait and multi-environment genomic prediction models were used (**Figure 7A**). MTME_CV2 and MT_CV2 models achieved the highest accuracies, with predictive abilities typically ranging from 0.74 to 0.77 on average across all sowing-by-season combinations (**Figure 7B**). The best-performing case corresponded to MT_CV2 applied to environment 2_2023–2024 (r = 0.8457), followed by MTME_CV2 in environment 2_2021–2022 (r = 0.8171). Other MTME_CV2 models associated with environments 1_2021–2022, 3_2021–2022, and 1_2022–2023 exhibited accuracies in the range of 0.78–0.80 (**Figure 7C**; **Supplementary Table 7**). These findings indicate that models integrating trait-level and environmental covariation capture the genetic determinants of SW effectively.

In contrast, single-environment and genomic-only (SE, G) models showed lower correlations (r = 0.36 on average), reflecting the limitations of environment-specific training. The inclusion of allelic information (G2) in ME and SE structures resulted in modest accuracy gains, particularly under ME_CV1_G2 and SE_G2 configurations. Predictive ability for the best models ranged from 0.85 to 0.54, with standard deviations spanning 0.05–0.11, indicating high reproducibility across folds and confirming the robustness of MT_CV2 and MTME_CV2 models.

### Predictive ability of GP models for morpho-phenological traits

The prediction of morpho-phenological (PH and HD) achieved exceptionally high accuracies (~0.9), particularly when using MTME models (**Supplementary Figures 2A**, **3A**; **Supplementary Tables 8**, **9**).

Regarding the HD prediction, the MTME_CV2 structure consistently delivered the highest predictive abilities, ranging from 0.90 to 0.97 with an average of 0.95 across all sowing-by-season combinations (**Supplementary Figure 2B**). The MTME_CV2 models once again dominated the top rankings, occupying the majority of the Top-20 positions (**Supplementary Figure 2C**; **Supplementary Table 8**). The best-performing case was MTME_CV2 for environment 2_2022–2023 (r = 0.9669), followed closely by MTME_CV2 applied to 2_2023–2024 (r = 0.9654) and 3_2022–2023 (r = 0.9594). These high accuracies, all above 0.95, confirm the remarkable predictive performance and reproducibility of MTME structures for HD.

These strong results indicate that HD, as a highly heritable phenological trait, can be predicted with very high accuracy using models that integrate both trait and environmental covariation. Multi-environment (ME_CV2_G2) models followed closely, achieving r = 0.92–0.94 across several environments (2_2023–2024, 2_2022–2023, 1_2022–2023, and 3_2022–2023). These results highlight the advantage of including allelic information (G2) to improve cross-environment prediction. In contrast, MT and SE models achieved lower but still strong accuracies (r = 0.81–0.82), indicating reliable prediction even under simplified structures, maintained solid performance levels, consistent with the high heritability of HD. Across all Top-20 models, predictive ability ranged from 0.97 to 0.81, with most cases exceeding 0.90, confirming HD as one of the most accurately predicted traits in the study, reflecting the exceptional stability and robustness of MTME_CV2 and ME_CV2_G2 models. The overall robustness and precision of MTME_CV2 highlights its effectiveness in predicting phenological traits such as heading date in complex breeding datasets.

Conversely, the prediction of the morphological trait (PH) performed remarkably well for all models, with the MTME model standing out by achieving the highest predictive accuracies (**Supplementary Figure 3B**). The MTME_CV2 framework was the dominant performer, reaching r = 0.9723 in environment 2_2022–2023, followed closely by MTME_CV2 models for 2_2021–2022 (r = 0.9698) and 1_2022–2023 (r = 0.9688). These results, all above 0.96, indicate exceptional predictive reliability and confirm that PH is a highly heritable and stable trait across environments (**Supplementary Figure 3C**; **Supplementary Table 9**). ME_CV2_G2 models followed closely behind, typically achieving r = 0.94–0.95 across multiple environments. Their strong performance demonstrates that the integration of allelic combinations (G2) adds genetic resolution and robustness to multi-environment predictions. Standard deviations remained low (0.01–0.03), confirming model stability across CV folds.

These results highlight the consistency of both MTME_CV2 and ME_CV2_G2 frameworks in capturing G×E patterns across sowing-by-season combinations. MTME and ME models clearly outperformed MT and SE approaches, even for instance MT_CV1_G2 and SE_G2 models yielded slightly lower but still strong accuracies (r = 0.89–0.91), showing that accounting for both genetic and environmental structure is critical for accurate prediction of PH. Simpler models such as MT_CV1_G2 and SE_G2 remained competitive, confirming that PH prediction is robust even under reduced model complexity.

Overall, these findings validate MTME_CV2 as the most effective and consistent approach for predicting both morpho-phenological traits across diverse sowing-by-season combinations and environmental conditions.

## Discussion

The present study provides an integrative assessment of multi-trait and multi-environment GP for durum wheat evaluated under different simulated Mediterranean conditions. By jointly modeling correlated traits and G×E interactions across several sowing-by-season combinations, this research demonstrates the clear advantage of complex prediction frameworks, particularly the MTME models implemented with CV2, for enhancing the prediction of yield-related and adaptive morpho-phenological traits. These results highlight the maturity of GS technology as a decision-support tool for durum breeding programs operating under climatic uncertainty and environmental heterogeneity.

### Superior performance of MTME models across traits and environments

Across all traits evaluated, MTME models consistently yielded the highest prediction accuracies, with performance gains of 5–20% over MT or ME models, and up to 40% relative to the SE baseline. The MTME framework leverages both genetic and environmental covariances, effectively borrowing information from correlated traits and environments (Montesinos-López et al., 2016, 2018b, 2018c, 2019a; Crossa et al., 2025a, 2025b). Such integrative modeling captures shared genetic determinants influencing multiple traits while accounting for plastic responses to environmental cues, thus providing a biologically realistic representation of durum wheat adaptation.

The improvement obtained under MTME-CV2 relative to CV1 reflects the model’s robustness in predicting genotype performance in unobserved environments for target lines, a critical feature for forward breeding and climate-resilient selection (Lopez-Cruz et al., 2015; Cuevas et al., 2018). Similar patterns have been reported in spring wheat (Sukumaran et al., 2017), maize (Jarquin et al., 2020), and barley (Bhatta et al., 2020), confirming that the joint exploitation of inter-trait and inter-environment covariance is a universally effective strategy across cereals. In durum wheat, where environments differ substantially between years and sites (e.g., Canada, Italy, Turkey, Mexico), these cross-links become even more relevant. Additionally, MT and MTME showed higher predictive performances, especially in CV2 designing. This is mostly due to the validation design used, which allows the inclusion of the performances of the target lines in some environments and of secondary and target traits in the training population, and predicts their target traits in other environments (testing), where the secondary traits are also available. This has widely been observed in previous studies (Lado et al., 2018; Vitale et al., 2024).

Finally, it is worth noting that while SE and ME analyses can be efficiently run on most commercial laptops, MT and, in particular, MTME analyses may be computationally demanding for such machines. Using a high-performance computing system, we observed a substantial increase in computational time for MT and MTME models compared with SE and ME, which were considerably faster.

### Trait-specific responses and biological interpretation

The magnitude of prediction accuracy varied among traits, reflecting their genetic architecture, heritability, and correlation with other traits.

High predictive ability (r > 0.81) with MTME-CV2 was observed for all five yield-related traits investigated. This suggests that the MTME model effectively disentangled additive and non-additive sources of variation across seasonal fluctuations in temperature and water availability. The simultaneous modeling likely benefited from their shared physiological pathways in sink-source allocation, as previously shown in multi-trait genomic studies in bread and durum wheat (Lado et al., 2018; Montesinos-López et al., 2019b). Their predictable behavior within the MTME framework is consistent with the genetic stability of these traits and their moderate-to-high heritability (Taranto et al., 2023).

Importantly, heading date (HD) and plant height (PH) reached the highest accuracies overall (r > 0.95), confirming their simpler genetic control and robust expression across environments. These traits are highly correlated with flowering time and photoperiod sensitivity genes (e.g., *Ppd-A1*, *Vrn-A1*), whose allelic effects were captured in the G2 matrix incorporating known functional variants (Puglisi et al., 2025).

Taken together, these results confirm that combining multiple traits within an MTME framework not only increases predictive accuracy but also improves the interpretability of genotype responses across time and space. In durum wheat, where yield is the emergent outcome of multiple interacting developmental traits, such multi-trait modeling mirrors the true biological complexity of performance.

### Integration of allelic matrices and functional genomics

An innovative aspect of this study is the inclusion of both the conventional genomic relationship matrix (G) and an allelic matrix (G2) capturing functional gene combinations. This dual approach allowed quantification of how allelic information at key loci complements genome-wide additive effects. As previously reported for the SE and ME GP models (Puglisi et al., 2025), the G2-based models improved the prediction accuracy for morpho-phenological traits by 2–4%, also when using MT and MEME approaches, suggesting that known allelic variants contribute unique information not captured by SNP-based relationships. This is consistent with recent reports integrating gene-level annotations and known regulatory regions into genomic prediction frameworks (Crossa et al., 2025a).

Moreover, although the improvement was modest compared to morpho-phenological traits, the use of G2 increased the prediction accuracy for yield-related traits. This outcome supports its stabilizing effect under multi-environment conditions and highlights the importance of jointly modeling allelic effects, environmental factors, and trait correlations. The limited improvement may reflect the need to further investigate genes with stronger effects on these traits (Nadolska-Orczyk et al., 2017).

In durum wheat, functional markers linked to *Ppd*, *Rht*, and *Vrn* genes have well-documented effects on developmental timing and architecture. Incorporating these alleles into GP models enhances prediction accuracy and biological interpretability, particularly under contrasting photothermal regimes typical of Mediterranean environments. Beyond developmental traits, the approach provides a blueprint for future integration of multi-omic data (e.g., transcriptomic, metabolomic, and spectral information) to capture both static and dynamic determinants of performance (Wang et al., 2019; Wu et al., 2022, 2024; Crossa et al., 2025a).

### Implications for durum wheat breeding pipelines

The clear superiority of MTME-CV2 models underlines the need to shift from traditional one-trait, one-environment evaluation pipelines to integrated, multi-layered prediction systems. In practical terms, breeders can use MTME predictions to (i) identify lines with consistent performance across environments, (ii) predict untested genotypes in new sowing cycles, and (iii) optimize selection indices incorporating correlated traits (e.g., GN, GW, SL). When combined with economic weights or multi-trait selection indices the MTME framework can directly inform decision-making to maximize multi-trait genetic gain per cycle.

Moreover, the high accuracy of different sowing date predictions supports the implementation of sowing-specific genomic selection, where training populations are updated annually with the latest phenotypic and environmental data. Such dynamic updating has been shown to accelerate genetic gain by maintaining model relevance under changing climate and management conditions (Crossa et al., 2017). For durum wheat breeding programs in the Mediterranean Basin, where season-to-season variability is pronounced, this adaptive strategy could significantly enhance selection efficiency and stability.

### Future directions: toward multi-modal and climate-smart prediction

While the present study focuses on genomic and phenotypic data, future expansions should integrate multi-modal inputs (e.g, environmental covariables, near-infrared spectra, time-series phenotyping data) to further improve model realism and predictive power. Multi-modal deep learning (MM-DL) frameworks (Montesinos-López et al., 2024; Crossa et al., 2025a, 2025b) can capture nonlinear relationships and trait-environment interactions that conventional kernels might overlook. For durum wheat, integrating environmental covariables aligned with phenological stages (e.g., sowing–heading, heading–flowering, flowering–harvest) has proven particularly valuable in previous CIMMYT studies (Basnet et al., 2019; Crossa et al., 2022; Cuevas et al., 2025).

Ultimately, the convergence of MTME statistical models and multi-modal data integration represents the frontier of prediction-based breeding. By combining the interpretability of kernel-based frameworks with the representational power of deep learning, breeders can more precisely identify genotypes with stable, high performance across variable environments—an essential requirement for ensuring food security in the face of climate change.

## Summary

This study demonstrates that multi-trait–multi-environment genomic prediction frameworks substantially enhance predictive ability for complex traits in durum wheat, especially under Mediterranean conditions characterized by strong environmental heterogeneity. The integration of allelic matrices, temporal grouping, and cross-validation strategies provides a robust foundation for predictive breeding. Beyond methodological innovation, these findings reaffirm the feasibility of predictive, data-driven breeding pipelines that transcend traditional selection cycles and directly target genotype performance in future environments.

By merging genomics, physiology, and environmental modeling, durum wheat breeding stands poised to achieve faster, more resilient genetic improvement, supporting sustainable pasta production and food security across the Mediterranean Basin and beyond.

## Data Availability

The original contributions presented in the study are included in the article/**Supplementary Material**. The SNP data are available in the Figshare database (https://figshare.com/) under the following DOI: 10.6084/m9.figshare.28554947 (Puglisi et al., 2025). The phenotypic data used in this study are also available in the Figshare database under the following DOI: 10.6084/m9.figshare.31702732. Further inquiries can be directed to the corresponding authors.

## References

[B1] Basnet B. R. Crossa J. Dreisigacker S. Pérez-Rodríguez P. Manes Y. Singh R. P. . (2019). Hybrid wheat prediction using genomic, pedigree, and environmental covariables interaction models. Plant Genome 12, 180051. doi: 10.3835/plantgenome2018.07.0051, PMID: 30951082 PMC12810112

[B2] Bates D. Mächler M. Bolker B. Walker S. (2015). Fitting linear mixed-effects models using lme4. J. Stat. Software 67, 1–48. doi: 10.18637/jss.v067.i01

[B3] Bhatta M. Gutierrez L. Cammarota L. Cardozo F. Germán S. Gómez-Guerrero B. . (2020). Multi-trait genomic prediction model increased the predictive ability for agronomic and malting quality traits in barley (Hordeum vulgare L.). G3: Genes Genomes Genet. 10, 1113–1124. doi: 10.1534/g3.119.400968, PMID: 31974097 PMC7056970

[B4] Crossa J. Cornelius P. L. Yan W. (2002). Biplots of linear-bilinear models for studying crossover genotype × Environment interaction. Crop Sci. 42, 619–633. doi: 10.2135/cropsci2002.6190

[B5] Crossa J. Martini J. W. R. Vitale P. Pérez-Rodríguez P. Costa-Neto G. Fritsche-Neto R. . (2025a). Expanding genomic prediction in plant breeding: harnessing big data, machine learning, and advanced software. Trends Plant Sci. 30, 1–19. doi: 10.1016/j.tplants.2024.12.009, PMID: 39890501

[B6] Crossa J. Montesinos-Lopez O. A. Costa-Neto G. Vitale P. Martini J. W. R. Runcie D. . (2025b). Machine learning algorithms translate big data into predictive breeding accuracy. Trends Plant Sci. 30, 167–184. doi: 10.1016/j.tplants.2024.09.011, PMID: 39462718

[B7] Crossa J. Montesinos-López O. A. Pérez-Rodríguez P. Costa-Neto G. Fritsche-Neto R. Ortiz R. . (2022). “ Genome and Environment Based Prediction ModelsPrediction models and Methods of Complex TraitsComplex traits Incorporating Genotype × Environment Interaction BT,”. Eds. Ahmadi N. Bartholomé J. Genomic Prediction of Complex Traits: Methods and Protocols ( Springer US, New York, NY), 245–283. doi: 10.1007/978-1-0716-2205-6_9, PMID: 35451779

[B8] Crossa J. Pérez-Rodríguez P. Cuevas J. Montesinos-López O. Jarquín D. de los Campos G. . (2017). Genomic selection in plant breeding: methods, models, and perspectives. Trends Plant Sci. 22, 961–975. doi: 10.1016/j.tplants.2017.08.011, PMID: 28965742

[B9] Cuevas J. Crossa J. Montesinos-lo A. Martini J. W. R. Sebastia G. Dreisigacker S. . (2025). Enhancing wheat genomic prediction by a hybrid kernel approach. Front. Plant Sci. 16, 1–15. doi: 10.3389/fpls.2025.1605202, PMID: 40838078 PMC12363290

[B10] Cuevas J. Crossa J. Montesinos-López O. A. Burgueño J. Pérez-Rodríguez P. de los Campos G. (2017). Bayesian genomic prediction with genotype × environment interaction kernel models. G3: Genes Genomes Genet. 7, 41–53. doi: 10.1534/g3.116.035584, PMID: 27793970 PMC5217122

[B11] Cuevas J. Crossa J. Soberanis V. Pérez-Elizalde S. Pérez-Rodríguez P. des los Campos G. . (2016). Genomic prediction of genotype × Environment interaction kernel regression models. Plant Genome 9, plantgenome2016.03.0024. doi: 10.3835/plantgenome2016.03.0024, PMID: 27902799

[B12] Cuevas J. Granato I. Fritsche-Neto R. Montesinos-Lopez O. A. Burgueño J. Sousa M. B. . (2018). Genomic-enabled prediction Kernel models with random intercepts for multi-environment trials. G3: Genes Genomes Genet. 8, 1347–1365. doi: 10.1534/g3.117.300454, PMID: 29476023 PMC5873923

[B13] Cuevas J. Reslow F. Crossa J. Ortiz R. (2023). Modeling genotype × environment interaction for single and multitrait genomic prediction in potato (Solanum tuberosum L.). G3 Genes|Genomes|Genetics 13, jkac322. doi: 10.1093/g3journal/jkac322, PMID: 36477309 PMC9911059

[B14] De Vita P. Taranto F. (2019). “ Durum Wheat (Triticum turgidum ssp. durum) Breeding to Meet the Challenge of Climate Change BT,”. Eds. Al-Khayri J. M. Jain S. M. Johnson D. V. Advances in Plant Breeding Strategies: Cereals. Volume 5 ( Springer International Publishing, Cham), 471–524. doi: 10.1007/978-3-030-23108-8_13, PMID:

[B15] Esposito S. Vitale P. Taranto F. Saia S. Pecorella I. D’Agostino N. . (2023). Simultaneous improvement of grain yield and grain protein concentration in durum wheat by using association tests and weighted GBLUP. Theor. Appl. Genet. 136, 1–21. doi: 10.1007/s00122-023-04487-8, PMID: 37947927

[B16] Hammami R. Sissons M. (2020). “ Durum wheat products, couscous BT,”. Eds. Igrejas G. Ikeda T. M. Guzmán C. Wheat Quality for Improving Processing and Human Health ( Springer International Publishing, Cham), 347–367. doi: 10.1007/978-3-030-34163-3_15, PMID:

[B17] Jarquín D. Crossa J. Lacaze X. Du Cheyron P. Daucourt J. Lorgeou J. . (2014). A reaction norm model for genomic selection using high-dimensional genomic and environmental data. Theor. Appl. Genet. 127, 595–607. doi: 10.1007/s00122-013-2243-1, PMID: 24337101 PMC3931944

[B18] Jarquin D. Howard R. Crossa J. Beyene Y. Gowda M. Martini J. W. R. . (2020). Genomic prediction enhanced sparse testing for multi-environment trials. G3: Genes Genomes Genet. 10, 2725–2739. doi: 10.1534/g3.120.401349, PMID: 32527748 PMC7407457

[B19] Jia Y. Jannink J. L. (2012). Multiple-trait genomic selection methods increase genetic value prediction accuracy. Genetics 192, 1513–1522. doi: 10.1534/genetics.112.144246, PMID: 23086217 PMC3512156

[B20] Lado B. Vázquez D. Quincke M. Silva P. Aguilar I. Gutiérrez L. (2018). Resource allocation optimization with multi-trait genomic prediction for bread wheat (Triticum aestivum L.) baking quality. Theor. Appl. Genet. 131, 2719–2731. doi: 10.1007/s00122-018-3186-3, PMID: 30232499 PMC6244535

[B21] Lopes M. S. Bovenhuis H. van Son M. Nordbø Ø. Grindflek E. H. Knol E. F. . (2017). Using markers with large effect in genetic and genomic predictions. J. Anim. Sci. 95, 59–71. doi: 10.2527/jas.2016.0754, PMID: 28177367

[B22] Lopez-Cruz M. Crossa J. Bonnett D. Dreisigacker S. Poland J. Jannink J. L. . (2015). Increased prediction accuracy in wheat breeding trials using a marker × environment interaction genomic selection model. G3: Genes Genomes Genet. 5, 569–582. doi: 10.1534/g3.114.016097, PMID: 25660166 PMC4390573

[B23] Martínez-Moreno F. Ammar K. Solis I. (2022). Global changes in cultivated area and breeding activities of durum wheat from 1800 to date: A historical review. Agronomy 12, 1–17. doi: 10.3390/agronomy12051135, PMID: 41725453

[B24] Medina C. A. Kaur H. Ray I. Yu L.-X. (2021). Strategies to increase prediction accuracy in genomic selection of complex traits in alfalfa (Medicago sativa L.). Cells 10. doi: 10.3390/cells10123372, PMID: 34943880 PMC8699225

[B25] Meuwissen T. H. E. Hayes B. J. Goddard M. E. (2001). Prediction of total genetic value using genome-wide dense marker maps. Genetics 157, 1819–1829. doi: 10.1093/genetics/157.4.1819, PMID: 11290733 PMC1461589

[B26] Montesinos-López A. Crespo-Herrera L. Dreisigacker S. Gerard G. Vitale P. Saint Pierre C. . (2024). Deep learning methods improve genomic prediction of wheat breeding. Front. Plant Sci. 15. doi: 10.3389/fpls.2024.1324090, PMID: 38504889 PMC10949530

[B27] Montesinos-López O. A. Montesinos-López A. Crossa J. Gianola D. Hernández-Suárez C. M. Martín-Vallejo J. (2018b). Multi-trait, multi-environment deep learning modeling for genomic-enabled prediction of plant traits. G3 Genes|Genomes|Genetics 8, 3829–3840. doi: 10.1534/g3.118.200728, PMID: 30291108 PMC6288830

[B28] Montesinos-López O. A. Montesinos-López A. Crossa J. Montesinos-López J. C. Mota-Sanchez D. Estrada-González F. . (2018c). Prediction of multiple-trait and multiple-environment genomic data using recommender systems. G3 Genes|Genomes|Genetics 8, 131–147. doi: 10.1534/g3.117.300309, PMID: 29097376 PMC5765342

[B29] Montesinos-López O. A. Montesinos-López A. Crossa J. Toledo F. H. Pérez-Hernández O. Eskridge K. M. . (2016). A genomic bayesian multi-trait and multi-environment model. G3 Genes|Genomes|Genetics 6, 2725–2744. doi: 10.1534/g3.116.032359, PMID: 27342738 PMC5015931

[B30] Montesinos-López A. Montesinos-López O. A. Gianola D. Crossa J. Hernández-Suárez C. M. (2018a). Multi-environment genomic prediction of plant traits using deep learners with dense architecture. G3 Genes|Genomes|Genetics 8, 3813–3828. doi: 10.1534/g3.118.200740, PMID: 30291107 PMC6288841

[B31] Montesinos-López O. A. Montesinos-López A. Luna-Vázquez F. J. Toledo F. H. Pérez-Rodríguez P. Lillemo M. . (2019a). An R package for bayesian analysis of multi-environment and multi-trait multi-environment data for genome-based prediction. G3 Genes|Genomes|Genetics 9, 1355–1369. doi: 10.1534/g3.119.400126, PMID: 30819822 PMC6505148

[B32] Montesinos-López O. A. Montesinos-López J. C. Montesinos-López A. Ramírez-Alcaraz J. M. Poland J. Singh R. . (2022). Bayesian multitrait kernel methods improve multienvironment genome-based prediction. G3 Genes|Genomes|Genetics 12, jkab406. doi: 10.1093/g3journal/jkab406, PMID: 34849802 PMC9210316

[B33] Montesinos-López O. A. Montesinos-López A. Tuberosa R. Maccaferri M. Sciara G. Ammar K. . (2019b). Multi-trait, multi-environment genomic prediction of durum wheat with genomic best linear unbiased predictor and deep learning methods. Front. Plant Sci. 10. doi: 10.3389/fpls.2019.01311, PMID: 31787990 PMC6856087

[B34] Nadolska-Orczyk A. Rajchel I. K. Orczyk W. Gasparis S. (2017). Major genes determining yield-related traits in wheat and barley. Theor. Appl. Genet. 130, 1081–1098. doi: 10.1007/s00122-017-2880-x, PMID: 28314933 PMC5440550

[B35] Pacheco Á. Vargas M. Alvarado G. Rodríguez F. S. Crossa J. Burgueño J. (2018). GEA-R (Genotype x Environment Analysis with R for Windows) Version 4.1. Available online at: https://api.semanticscholar.org/CorpusID:125914150 (Accessed February 5, 2026).

[B36] Pérez P. de los Campos G. (2014). BGLR: A statistical package for whole genome regression and prediction. Genetics 198, 483–495. doi: 10.1534/genetics.114.164442, PMID: 25009151 PMC4196607

[B37] Pérez-Rodríguez P. de los Campos G. (2022). Multitrait Bayesian shrinkage and variable selection models with the BGLR-R package. Genetics 222. doi: 10.1093/genetics/iyac112, PMID: 35924977 PMC9434216

[B38] Puglisi D. Afshari-behbahanizadeh S. Angione G. Colella I. Fania F. Spadanuda P. . (2025). Genomic prediction models for morpho-phenological traits in durum wheat based on Vrn, Ppd, and Rht alleles. Plant Genome 14, 1–23. doi: 10.1002/tpg2.70072, PMID: 41257385 PMC12628358

[B39] Puglisi D. Delbono S. Visioni A. Ozkan H. Kara İ. Casas A. M. . (2021). Genomic prediction of grain yield in a barley MAGIC population modeling genotype per environment interaction. Front. Plant Sci. 12. doi: 10.3389/fpls.2021.664148, PMID: 34108982 PMC8183822

[B40] R Core Team (2020). R: A language and environment for statistical computing (Vienna: R Foundation for Statistical Computing). Available online at: https://www.r-project.org/ (Accessed February 5, 2026).

[B41] Samonte S. O. P. B. Wilson L. T. McClung A. M. Medley J. C. (2005). Targeting cultivars onto rice growing environments using AMMI and SREG GGE biplot analyses. Crop Sci. 45, 2414–2424. doi: 10.2135/cropsci2004.0627

[B42] Schloerke B. Cook D. Briatte F. Marbach M. Thoen E. . (2025). GGally: Extension to “ggplot2” R package version 2.4.0. Available online at: https://ggobi.github.io/ggally/ (Accessed February 5, 2026).

[B43] Su G. Christensen O. F. Janss L. Lund M. S. (2014). Comparison of genomic predictions using genomic relationship matrices built with different weighting factors to account for locus-specific variances. J. Dairy Sci. 97, 6547–6559. doi: 10.3168/jds.2014-8210, PMID: 25129495

[B44] Sukumaran S. Crossa J. Jarquin D. Lopes M. Reynolds M. P. (2017). Genomic prediction with pedigree and genotype × Environment interaction in spring wheat grown in south and west asia, north africa, and Mexico. G3 Genes|Genomes|Genetics 7, 481–495. doi: 10.1534/g3.116.036251, PMID: 27903632 PMC5295595

[B45] Taranto F. Esposito S. De Vita P. (2023). Genomics for yield and yield components in durum wheat. Plants 12, 1–19. doi: 10.3390/plants12132571, PMID: 37447132 PMC10346609

[B46] VanRaden P. M. (2008). Efficient methods to compute genomic predictions. J. Dairy Sci. 91, 4414–4423. doi: 10.3168/jds.2007-0980, PMID: 18946147

[B47] Vitale P. Laidò G. Dono G. Pecorella I. Ramasubramanian V. Lorenz A. . (2024). Univariate and multivariate genomic prediction for agronomic traits in durum wheat under two field conditions. PloS One 19, e0310886. doi: 10.1371/journal.pone.0310886, PMID: 39541330 PMC11563401

[B48] Wang S. Wei J. Li R. Qu H. Chater J. M. Ma R. . (2019). Identification of optimal prediction models using multi-omic data for selecting hybrid rice. Heredity 123, 395–406. doi: 10.1038/s41437-019-0210-6, PMID: 30911139 PMC6781126

[B49] Wickham H. (2016). ggplot2: Elegant Graphics for Data Analysis (New York: Springer-Verlag). doi: 10.1007/978-0-387-98141-3, PMID:

[B50] Wu C. Luo J. Xiao Y. (2024). Multi-omics assists genomic prediction of maize yield with machine learning approaches. Mol. Breed. 44, 14. doi: 10.1007/s11032-024-01454-z, PMID: 38343399 PMC10853138

[B51] Wu P.-Y. Stich B. Weisweiler M. Shrestha A. Erban A. Westhoff P. . (2022). Improvement of prediction ability by integrating multi-omic datasets in barley. BMC Genomics 23, 200. doi: 10.1186/s12864-022-08337-7, PMID: 35279073 PMC8917753

[B52] Zadoks J. C. Chang T. T. Konzak C. F. (1974). A decimal code for the growth stages of cereals. World Pumps 14, 415–421. doi: 10.1016/s0262-1762(99)80614-2, PMID: 41674266

