# Correction: Multi-trait and multi-environment genomic prediction enhances yield components improvement in durum wheat

**DOI:** 10.3389/fpls.2026.1832903

**Published:** 2026-04-06

**Authors:** Damiano Puglisi, José Crossa, Jaime Cuevas, Fabio Fania, Sanaz Afshari-Behbahanizadeh, Paolo Vitale, Pasquale De Vita

**Affiliations:** 1 CREA – Research Centre for Cereal and Industrial Crops (CREA-CI) Consiglio per la Ricerca in Agricoltura e l’Analisi dell’Economia Agraria, Foggia, Italy; 2 Colegio de Postgraduados, Texcoco, Estado de México, Mexico; 3 Division de Ciencias, Ingeniería y Tecnología (DCIT), Universidad Autónoma del Estado de Quintana Roo, Chetumal, Quintana Roo, Mexico; 4 Department of Agriculture, Food, Natural Science, Engineering (DAFNE), University of Foggia, Foggia, Italy; 5 International Maize and Wheat Improvement Center (CIMMYT), Texcoco, Estado de México, Mexico

**Keywords:** gene-based relationship matrix, genomic selection, GxE interaction, SNP markers, sowing time, yield-related traits

In the published article, there was an error in the author list, and author Sanaz Afshari-Behbahanizadeh was erroneously excluded, due to an oversight during the data acquisition and manuscript preparation stages. The corrected author list appears below.

Damiano Puglisi^1^, José Crossa^2^, Jaime Cuevas^3^, Fabio Fania^1^, Sanaz Afshari-Behbahanizadeh^1,4^, Paolo Vitale^5*^, Pasquale De Vita^1*^

A correction has been made to the **Author Contributions**. This section previously stated:

“DP: Writing – original draft, Data curation, Conceptualization, Visualization, Writing – review & editing, Methodology. JCr: Software, Supervision, Writing – review & editing, Writing – original draft, Methodology. JCu: Writing – review & editing, Software. FF: Data curation, Writing – review & editing. PV: Software, Writing – review & editing, Conceptualization, Writing – original draft, Methodology. PD: Funding acquisition, Resources, Conceptualization, Supervision, Writing – review & editing”.

The **Author Contributions** Statement has been corrected to read:

“DP: Writing – original draft, Data curation, Conceptualization, Visualization, Writing – review & editing, Methodology. JCr: Software, Supervision, Writing – review & editing, Writing – original draft, Methodology. JCu: Writing – review & editing, Software. FF: Data curation, Writing – review & editing. SA-B: Data curation, Writing – review & editing. PV: Software, Writing – review & editing, Conceptualization, Writing – original draft, Methodology. PD: Funding acquisition, Resources, Conceptualization, Supervision, Writing – review & editing”.

In the published article, there was an omission in the **Data Availability Statement** regarding the availability of the phenotypic data used for the analyses. This correction does not involve the addition of new data to the study but provides the link to the existing data required to support the published results.

A correction has been made to the **Data Availability Statement**. This statement previously stated:

“The original contributions presented in the study are included in the article/supplementary material. The SNP data are available in the Figshare database (https://figshare.com/) under the following DOI: 10.6084/m9.figshare.28554947 (Puglisi et al., 2025). Further inquiries can be directed to the corresponding authors”.

The corrected **Data Availability Statement** appears below:

“The original contributions presented in the study are included in the article/supplementary material. The SNP data are available in the Figshare database (https://figshare.com/) under the following DOI: 10.6084/m9.figshare.28554947 (Puglisi et al., 2025). The phenotypic data used in this study are also available in the Figshare database under the following DOI: 10.6084/m9.figshare.31702732. Further inquiries can be directed to the corresponding authors”.

The original article has been updated.

