# Supplementary material for: Multi-trait and multi-environment genomic prediction enhances yield components improvement in durum wheat
Source: Front Plant Sci. 2026 Feb 23;17:1759897. doi: 10.3389/fpls.2026.1759897 (PMC12968193; doi:10.3389/fpls.2026.1759897)
Supplement: Supplementary file 1 [file DataSheet1.pdf]

Multi-trait and Multi-environment Genomic Prediction Enhances Yield Components Improvement in Durum Wheat

D. Puglisi[1], J. Crossa[2], J. Cuevas[3], F. Fania[1], P. Vitale[4], and P. De Vita[1]

[1]CREA – Research Centre for Cereal and Industrial Crops (CREA-CI) Consiglio per la Ricerca in Agricoltura e l'Analisi dell'Economia Agraria S.S. 673, m 25,200 71122 Foggia (FG), Italy
[2]Colegio de Postgraduados, Campus Montecillo, Carretera México-Texcoco, Km. 36.5, Montecillo, Texcoco, Estado de México C.P. 56264, México
[3]Division de Ciencias, Ingeniería y Tecnología (DCIT), Universidad Autónoma del Estado de Quintana Roo, Chetumal, Quintana Roo, 77019, México
[4]International Maize and Wheat Improvement Center (CIMMYT) Carretera México-Veracruz, Km. 45, El Batán, Texcoco, Estado de México 56237 México

Supplementary Tables

Supplementary Table 1. Panel of 186 durum wheat genotypes used in this study. For each ID genotype, year, country, type [landraces (LR), old cultivars (OC), modern cultivars (MC)], and the allelic variation at *Vrn-A1*, *Ppd-A1*, *Ppd-B1*, and *Rht-B1* genes were shown.

**ID**	**Genotype**	**Year**	**Country**	**Type**	***Vrn-A1***	***Ppd-A1***	***Ppd-B1***	***Rht-B1***
G001	Achille	2006	Italy	MC	*Vrn-A1c*	*Ppd-A1b*	*Ppd-B1b*	*Rht-B1b*
G002	Ambral	1986	France	MC	*Vrn-A1b*	*Ppd-A1b*	*Ppd-B1b*	*Rht-B1b*
G003	Ancomarzio	2003	Italy	MC	*Vrn-A1c*	*Ppd-A1a(GS105)*	*Ppd-B1a*	*Rht-B1b*
G004	Antalis	2013	Italy	MC	*Vrn-A1c*	*Ppd-A1a(GS105)*	*Ppd-B1a*	*Rht-B1b*
G005	Antas	1988	Italy	MC	*Vrn-A1c*	*Ppd-A1b*	*Ppd-B1a*	*Rht-B1b*
G006	Anvergur	n.a.	France	MC	*Vrn-A1c*	*Ppd-A1b*	*Ppd-B1b*	*Rht-B1b*
G007	Appulo	1973	Italy	OC	*Vrn-A1c*	*Ppd-A1b*	*Ppd-B1a*	*Rht-B1a*
G008	Arcangelo	1983	Italy	MC	*Vrn-A1c*	*Ppd-A1b*	*Ppd-B1b*	*Rht-B1b*
G009	Ariosto	2005	Italy	MC	*Vrn-A1*	*Ppd-A1b*	*Ppd-B1b*	*Rht-B1b*
G010	Athena	1982	Italy	MC	*Vrn-A1c*	*Ppd-A1b*	*Ppd-B1b*	*Rht-B1b*
G011	Atoudur	n.a.	France	MC	*Vrn-A1c*	*Ppd-A1b*	*Ppd-B1a*	*Rht-B1b*
G012	Aureo	2009	Italy	MC	*Vrn-A1c*	*Ppd-A1a(GS105)*	*Ppd-B1b*	*Rht-B1b*
G013	Avispa	2001	Italy	MC	*Vrn-A1c*	*Ppd-A1a(GS105)*	*Ppd-B1b*	*Rht-B1b*
G014	Aziziah	1933	Italy	OC	*Vrn-A1c*	*Ppd-A1b*	*Ppd-B1a*	*Rht-B1b*
G015	Aziziah	1925	Italy	OC	*Vrn-A1b*	*Ppd-A1b*	*Ppd-B1b*	*Rht-B1a*
G016	Aziziah_301	n.a.	Italy	OC	*Vrn-A1c*	*Ppd-A1b*	*Ppd-B1b*	*Rht-B1a*
G017	Aziziah	1929	Italy	OC	*Vrn-A1c*	*Ppd-A1b*	*Ppd-B1a*	*Rht-B1b*
G018	B_52	1966	Italy	OC	*Vrn-A1c*	*Ppd-A1b*	*Ppd-B1a*	*Rht-B1a*
G019	Babylone	n.a.	France	MC	*Vrn-A1c*	*Ppd-A1b*	*Ppd-B1a*	*Rht-B1b*
G020	Belfuggito	1973	Italy	OC	*Vrn-A1*	*Ppd-A1b*	*Ppd-B1b*	*Rht-B1a*
G021	Belsincap_8	1966	Italy	OC	*Vrn-A1c*	*Ppd-A1b*	*Ppd-B1a*	*Rht-B1a*
G022	Beltorax	2017	Italy	MC	*Vrn-A1*	*Ppd-A1a(GS105)*	*Ppd-B1a*	*Rht-B1b*
G023	Biancuccia	1930	Italy	LR	*Vrn-A1c*	*Ppd-A1b*	*Ppd-B1b*	*Rht-B1a*
G024	Biensur	n.a.	France	MC	*Vrn-A1c*	*Ppd-A1b*	*Ppd-B1a*	*Rht-B1b*
G025	Bufala_Nera_Lunga	n.a.	Italy	LR	*Vrn-A1b*	*Ppd-A1b*	*Ppd-B1a*	*Rht-B1a*
G026	Cannizzara	n.a.	Italy	LR	*Vrn-A1*	*Ppd-A1b*	*Ppd-B1b*	*Rht-B1a*
G027	Canu_Bascia	n.a.	Italy	LR	*Vrn-A1*	*Ppd-A1b*	*Ppd-B1b*	*Rht-B1a*
G028	Capeiti_8	n.a.	Italy	OC	*Vrn-A1c*	*Ppd-A1b*	*Ppd-B1b*	*Rht-B1a*
G029	Capinera	n.a.	Italy	LR	*Vrn-A1c*	*Ppd-A1b*	*Ppd-B1a*	*Rht-B1a*
G030	Cappelli	1915	Italy	OC	*Vrn-A1b*	*Ppd-A1b*	*Ppd-B1a*	*Rht-B1a*
G031	Cappelli	1915	Italy	OC	*Vrn-A1b*	*Ppd-A1b*	*Ppd-B1b*	*Rht-B1a*
G032	Cappelli	1915	Italy	OC	*Vrn-A1b*	*Ppd-A1b*	*Ppd-B1b*	*Rht-B1a*
G033	Carlantino	n.a.	Italy	LR	*Vrn-A1c*	*Ppd-A1b*	*Ppd-B1b*	*Rht-B1a*
G034	Carlo_Yucci	n.a.	Italy	OC	*Vrn-A1*	*Ppd-A1b*	*Ppd-B1a*	*Rht-B1a*
G035	Casale_92	n.a.	Italy	OC	*Vrn-A1c*	*Ppd-A1b*	*Ppd-B1b*	*Rht-B1a*
G036	Castel_Del_Monte	1969	Italy	OC	*Vrn-A1c*	*Ppd-A1b*	*Ppd-B1b*	*Rht-B1a*
G037	Castello	1987	Italy	MC	*Vrn-A1c*	*Ppd-A1b*	*Ppd-B1a*	*Rht-B1b*
G038	CastelPorziano_MG3713	1969	Italy	OC	*Vrn-A1b*	*Ppd-A1b*	*Ppd-B1a*	*Rht-B1a*
G039	CastelPorziano_MG7245	1969	Italy	OC	*Vrn-A1b*	*Ppd-A1b*	*Ppd-B1a*	*Rht-B1a*
G040	Catasta	2009	Italy	MC	*Vrn-A1c*	*Ppd-A1b*	*Ppd-B1b*	*Rht-B1b*
G041	Ciccio	1996	Italy	MC	*Vrn-A1c*	*Ppd-A1b*	*Ppd-B1a*	*Rht-B1b*
G042	Ciclope_66	1966	Italy	OC	*Vrn-A1b*	*Ppd-A1b*	*Ppd-B1a*	*Rht-B1a*
G043	Ciclope	2006	Italy	MC	*Vrn-A1c*	*Ppd-A1b*	*Ppd-B1b*	*Rht-B1b*
G044	Claudio	1998	Italy	MC	*Vrn-A1*	*Ppd-A1a(GS105)*	*Ppd-B1b*	*Rht-B1b*
G045	Cliodur	2014	n.a.	MC	*Vrn-A1b*	*Ppd-A1b*	*Ppd-B1a*	*Rht-B1a*
G046	Colosseo	1995	Italy	MC	*Vrn-A1c*	*Ppd-A1a(GS105)*	*Ppd-B1b*	*Rht-B1b*
G047	Core	2008	Italy	MC	*Vrn-A1c*	*Ppd-A1a(GS105)*	*Ppd-B1b*	*Rht-B1b*
G048	Creso	1974	Italy	MC	*Vrn-A1c*	*Ppd-A1b*	*Ppd-B1b*	*Rht-B1b*
G049	Dauno_III4879	1914	Italy	OC	*Vrn-A1c*	*Ppd-A1b*	*Ppd-B1b*	*Rht-B1a*
G050	Dauno_III	1914	Italy	OC	*Vrn-A1c*	*Ppd-A1b*	*Ppd-B1b*	*Rht-B1a*

G051	Dauno_III	1914	Italy	OC	*Vrn-A1c*	*Ppd-A1b*	*Ppd-B1b*	*Rht-B1a*
G052	Dauno	1914	Italy	OC	*Vrn-A1c*	*Ppd-A1b*	*Ppd-B1a*	*Rht-B1a*
G053	Dauno	1914	Italy	OC	*Vrn-A1c*	*Ppd-A1b*	*Ppd-B1a*	*Rht-B1a*
G054	Dauno_III	1914	Italy	OC	*Vrn-A1c*	*Ppd-A1b*	*Ppd-B1b*	*Rht-B1a*
G055	Dauno_III	1914	Italy	OC	*Vrn-A1c*	*Ppd-A1b*	*Ppd-B1b*	*Rht-B1a*
G056	Decimomanna	n.a.	Italy	LR	*Vrn-A1c*	*Ppd-A1b*	*Ppd-B1b*	*Rht-B1a*
G057	Duilio	1984	Italy	MC	*Vrn-A1c*	*Ppd-A1a(GS105)*	*Ppd-B1a*	*Rht-B1b*
G058	Duro_De_Naples	n.a.	Italy	OC	*Vrn-A1b*	*Ppd-A1b*	*Ppd-B1a*	*Rht-B1a*
G059	Duro_SG_3	n.a.	Italy	OC	*Vrn-A1*	*Ppd-A1b*	*Ppd-B1a*	*Rht-B1a*
G060	Durobonus	n.a.	n.a.	MC	*Vrn-A1*	*Ppd-A1b*	*Ppd-B1a*	*Rht-B1b*
G061	Emilio_Lepido	2011	Italy	MC	*Vrn-A1c*	*Ppd-A1a(GS105)*	*Ppd-B1a*	*Rht-B1b*
G062	Ettore	2012	Italy	MC	*Vrn-A1*	*Ppd-A1a(GS105)*	*Ppd-B1b*	*Rht-B1b*
G063	Farro_Corto	n.a.	Italy	LR	*Vrn-A1*	*Ppd-A1b*	*Ppd-B1a*	*Rht-B1a*
G064	Farro_Lungo	n.a.	Italy	LR	*Vrn-A1b*	*Ppd-A1b*	*Ppd-B1b*	*Rht-B1a*
G065	Fiore	2001	Italy	MC	*Vrn-A1c*	*Ppd-A1a(GS105)*	*Ppd-B1b*	*Rht-B1b*
G066	Fortore	1995	Italy	MC	*Vrn-A1c*	*Ppd-A1b*	*Ppd-B1a*	*Rht-B1b*
G067	G_Miraglia	n.a.	Italy	OC	*Vrn-A1*	*Ppd-A1b*	*Ppd-B1b*	*Rht-B1a*
G068	Garigliano	1927	Italy	OC	*Vrn-A1c*	*Ppd-A1b*	*Ppd-B1a*	*Rht-B1a*
G069	Garigliano	1927	Italy	OC	*Vrn-A1c*	*Ppd-A1b*	*Ppd-B1a*	*Rht-B1a*
G070	Giustalisa	n.a.	Italy	LR	*Vrn-A1*	*Ppd-A1b*	*Ppd-B1b*	*Rht-B1a*
G071	GKJulidur	2014	n.a.	MC	*Vrn-A1*	*Ppd-A1b*	*Ppd-B1a*	*Rht-B1b*
G072	GKSelyemur	2014	n.a.	MC	*Vrn-A1b*	*Ppd-A1b*	*Ppd-B1a*	*Rht-B1b*
G073	Grazia	1985	Italy	MC	*Vrn-A1c*	*Ppd-A1b*	*Ppd-B1a*	*Rht-B1b*
G074	Grifoni235	1949	Italy	OC	*Vrn-A1b*	*Ppd-A1b*	*Ppd-B1b*	*Rht-B1a*
G075	Grifoni235	1949	Italy	OC	*Vrn-A1b*	*Ppd-A1b*	*Ppd-B1b*	*Rht-B1a*
G076	Grifoni	1949	Italy	OC	*Vrn-A1c*	*Ppd-A1b*	*Ppd-B1b*	*Rht-B1a*
G077	Grifoni235	1949	Italy	OC	*Vrn-A1c*	*Ppd-A1b*	*Ppd-B1b*	*Rht-B1a*
G078	Grifoni235	1949	Italy	OC	*Vrn-A1c*	*Ppd-A1b*	*Ppd-B1b*	*Rht-B1a*
G079	Hymera	1970	Italy	OC	*Vrn-A1c*	*Ppd-A1b*	*Ppd-B1b*	*Rht-B1a*
G080	Iride	1996	Italy	MC	*Vrn-A1c*	*Ppd-A1a(GS105)*	*Ppd-B1b*	*Rht-B1b*
G081	Ittu	n.a.	n.a.	MC	*Vrn-A1b*	*Ppd-A1b*	*Ppd-B1a*	*Rht-B1b*
G082	Jeanh_Rhetifha	n.a.	n.a.	LR	*Vrn-A1c*	*Ppd-A1b*	*Ppd-B1b*	*Rht-B1a*
G083	Jenah_Kottifa	n.a.	n.a.	LR	*Vrn-A1b*	*Ppd-A1b*	*Ppd-B1a*	*Rht-B1a*
G084	Kamut_Khorasan	n.a.	n.a.	LR	*Vrn-A1b*	*Ppd-A1b*	*Ppd-B1b*	*Rht-B1a*
G085	Kamut_Khorasan M_D	n.a.	n.a.	LR	*Vrn-A1b*	*Ppd-A1b*	*Ppd-B1b*	*Rht-B1a*
G086	Kanakis	2008	Italy	MC	*Vrn-A1c*	*Ppd-A1a(GS105)*	*Ppd-B1a*	*Rht-B1b*
G087	Kiperounda	n.a.	Marocco	OC	*Vrn-A1*	*Ppd-A1b*	*Ppd-B1b*	*Rht-B1a*
G088	L2284	n.a.	n.a.	MC	*Vrn-A1c*	*Ppd-A1b*	*Ppd-B1a*	*Rht-B1b*
G089	L2300_Natal	2016	n.a.	MC	*Vrn-A1c*	*Ppd-A1b*	*Ppd-B1b*	*Rht-B1b*
G090	Lambro	n.a.	Italy	OC	*Vrn-A1*	*Ppd-A1b*	*Ppd-B1b*	*Rht-B1a*
G091	Langdon	1956	United States	OC	*Vrn-A1b*	*Ppd-A1b*	*Ppd-B1a*	*Rht-B1a*
G092	Latinur	2004	France	MC	*Vrn-A1c*	*Ppd-A1a(GS105)*	*Ppd-B1b*	*Rht-B1b*
G093	Lesina	1998	n.a.	MC	*Vrn-A1c*	*Ppd-A1b*	*Ppd-B1a*	*Rht-B1b*
G094	Levante	2002	Italy	MC	*Vrn-A1c*	*Ppd-A1b*	*Ppd-B1a*	*Rht-B1b*
G095	LG_Anibis	n.a.	n.a.	MC	*Vrn-A1c*	*Ppd-A1a(GS105)*	*Ppd-B1b*	*Rht-B1b*
G096	Lloyd	1995	USA	MC	*Vrn-A1c*	*Ppd-A1b*	*Ppd-B1a*	*Rht-B1b*
G097	Logidur	n.a.	n.a.	MC	*Vrn-A1b*	*Ppd-A1b*	*Ppd-B1a*	*Rht-B1b*
G098	Lupidur	n.a.	n.a.	MC	*Vrn-A1*	*Ppd-A1b*	*Ppd-B1a*	*Rht-B1b*
G099	Maestrale	2004	Italy	MC	*Vrn-A1c*	*Ppd-A1a(GS105)*	*Ppd-B1b*	*Rht-B1b*
G100	Manto_Di_Maria	n.a.	n.a.	LR	*Vrn-A1c*	*Ppd-A1b*	*Ppd-B1b*	*Rht-B1a*
G101	Marakas	2012	n.a.	MC	*Vrn-A1*	*Ppd-A1a(GS105)*	*Ppd-B1b*	*Rht-B1b*
G102	Marco_Aurelio	2010	Italy	MC	*Vrn-A1c*	*Ppd-A1b*	*Ppd-B1b*	*Rht-B1b*
G103	Margherito	n.a.	Italy	LR	*Vrn-A1*	*Ppd-A1b*	*Ppd-B1a*	*Rht-B1a*
G104	Martinella	n.a.	Italy	LR	*Vrn-A1c*	*Ppd-A1b*	*Ppd-B1b*	*Rht-B1a*

G105	Martondur3	n.a.	n.a.	MC	*Vrn-A1*	*Ppd-A1b*	*Ppd-B1b*	*Rht-B1b*
G106	Marzellina_Fortore	n.a.	n.a.	LR	*Vrn-A1c*	*Ppd-A1b*	*Ppd-B1b*	*Rht-B1a*
G107	MarzellinaSaccone	n.a.	n.a.	LR	*Vrn-A1c*	*Ppd-A1b*	*Ppd-B1b*	*Rht-B1a*
G108	Massimo_meridio	2010	Italy	MC	*Vrn-A1c*	*Ppd-A1b*	*Ppd-B1b*	*Rht-B1b*
G109	Meridiano	1999	Italy	MC	*Vrn-A1c*	*Ppd-A1a(GS105)*	*Ppd-B1a*	*Rht-B1b*
G110	MG54	n.a.	Italy	MC	*Vrn-A1c*	*Ppd-A1b*	*Ppd-B1b*	*Rht-B1b*
G111	Mida	1975	Italy	MC	*Vrn-A1c*	*Ppd-A1b*	*Ppd-B1b*	*Rht-B1b*
G112	Miradoux	n.a.	France	MC	*Vrn-A1*	*Ppd-A1b*	*Ppd-B1a*	*Rht-B1b*
G113	Monastir	2009	Italy	MC	*Vrn-A1c*	*Ppd-A1b*	*Ppd-B1a*	*Rht-B1b*
G114	Murru	n.a.	Italy	LR	*Vrn-A1b*	*Ppd-A1b*	*Ppd-B1a*	*Rht-B1a*
G115	MVMakaroni	n.a.	n.a.	MC	*Vrn-A1*	*Ppd-A1b*	*Ppd-B1a*	*Rht-B1b*
G116	Nadif	2016	Italy	MC	*Vrn-A1*	*Ppd-A1a(GS105)*	*Ppd-B1b*	*Rht-B1b*
G117	Neodur	1987	France	MC	*Vrn-A1c*	*Ppd-A1b*	*Ppd-B1a*	*Rht-B1b*
G118	Normanno	2002	Italy	MC	*Vrn-A1c*	*Ppd-A1b*	*Ppd-B1a*	*Rht-B1b*
G119	Odessa66	n.a.	n.a.	MC	*Vrn-A1b*	*Ppd-A1a(GS105)*	*Ppd-B1b*	*Rht-B1b*
G120	Ofanto	1990	n.a.	MC	*Vrn-A1c*	*Ppd-A1b*	*Ppd-B1a*	*Rht-B1b*
G121	Opera	2013	n.a.	MC	*Vrn-A1c*	*Ppd-A1a(GS105)*	*Ppd-B1b*	*Rht-B1b*
G122	Orobel	1999	Italy	MC	*Vrn-A1c*	*Ppd-A1b*	*Ppd-B1a*	*Rht-B1b*
G123	Panoramix	2017	Italy	MC	*Vrn-A1c*	*Ppd-A1a(GS105)*	*Ppd-B1b*	*Rht-B1b*
G124	Pavone	n.a.	Italy	LR	*Vrn-A1c*	*Ppd-A1b*	*Ppd-B1a*	*Rht-B1a*
G125	Pietrafitta	1999	Italy	MC	*Vrn-A1c*	*Ppd-A1b*	*Ppd-B1a*	*Rht-B1b*
G126	Platani	1995	Italy	MC	*Vrn-A1c*	*Ppd-A1b*	*Ppd-B1b*	*Rht-B1b*
G127	Poggio	1997	Italy	MC	*Vrn-A1c*	*Ppd-A1b*	*Ppd-B1a*	*Rht-B1b*
G128	Pr22D89	2005	Italy	MC	*Vrn-A1c*	*Ppd-A1b*	*Ppd-B1a*	*Rht-B1b*
G129	Preco	1995	Italy	MC	*Vrn-A1c*	*Ppd-A1a(GS100)*	*Ppd-B1a*	*Rht-B1b*
G130	Prowidur	n.a.	n.a.	MC	*Vrn-A1b*	*Ppd-A1b*	*Ppd-B1b*	*Rht-B1b*
G131	Quattrocoste_Razza_148	n.a.	Italy	LR	*Vrn-A1c*	*Ppd-A1b*	*Ppd-B1b*	*Rht-B1a*
G132	Ramirez	2007	Italy	MC	*Vrn-A1c*	*Ppd-A1a(GS105)*	*Ppd-B1b*	*Rht-B1b*
G133	Ramirez	2007	Italy	MC	*Vrn-A1c*	*Ppd-A1a(GS105)*	*Ppd-B1b*	*Rht-B1b*
G134	Realforte	n.a.	Italy	LR	*Vrn-A1c*	*Ppd-A1b*	*Ppd-B1b*	*Rht-B1a*
G135	Relief	n.a.	n.a.	MC	*Vrn-A1c*	*Ppd-A1b*	*Ppd-B1a*	*Rht-B1b*
G136	Rgt_Daurur	2013	n.a.	MC	*Vrn-A1c*	*Ppd-A1b*	*Ppd-B1a*	*Rht-B1b*
G137	Roseta	n.a.	n.a.	LR	*Vrn-A1c*	*Ppd-A1b*	*Ppd-B1b*	*Rht-B1a*
G138	Rosso	n.a.	Italy	LR	*Vrn-A1c*	*Ppd-A1b*	*Ppd-B1a*	*Rht-B1a*
G139	Russello1_13	n.a.	Italy	LR	*Vrn-A1c*	*Ppd-A1b*	*Ppd-B1b*	*Rht-B1a*
G140	Russello2_14	n.a.	Italy	LR	*Vrn-A1c*	*Ppd-A1b*	*Ppd-B1b*	*Rht-B1a*
G141	Russello3_14	n.a.	Italy	LR	*Vrn-A1c*	*Ppd-A1b*	*Ppd-B1b*	*Rht-B1a*
G142	Russello5_26	n.a.	Italy	LR	*Vrn-A1c*	*Ppd-A1b*	*Ppd-B1b*	*Rht-B1a*
G143	Russello9	n.a.	Italy	LR	*Vrn-A1c*	*Ppd-A1b*	*Ppd-B1b*	*Rht-B1a*
G144	Russello	n.a.	Italy	LR	*Vrn-A1c*	*Ppd-A1b*	*Ppd-B1b*	*Rht-B1a*
G145	Saintly	n.a.	Australia	MC	*Vrn-A1c*	*Ppd-A1a(GS100)*	*Ppd-B1b*	*Rht-B1b*
G146	Sancarlo	1986	Italy	MC	*Vrn-A1c*	*Ppd-A1b*	*Ppd-B1a*	*Rht-B1b*
G147	Sansone	1977	Italy	MC	*Vrn-A1c*	*Ppd-A1a(GS105)*	*Ppd-B1a*	*Rht-B1b*
G148	Saragolla	2004	Italy	MC	*Vrn-A1c*	*Ppd-A1b*	*Ppd-B1a*	*Rht-B1b*
G149	Saragolla_Zingaresca	n.a.	Italy	LR	*Vrn-A1b*	*Ppd-A1b*	*Ppd-B1b*	*Rht-B1a*
G150	Saragolla	n.a.	Italy	LR	*Vrn-A1b*	*Ppd-A1b*	*Ppd-B1b*	*Rht-B1a*
G151	Saragolla_Rossa_B	n.a.	Italy	LR	*Vrn-A1c*	*Ppd-A1b*	*Ppd-B1a*	*Rht-B1a*
G152	Scorsonera	n.a.	Italy	LR	*Vrn-A1c*	*Ppd-A1b*	*Ppd-B1a*	*Rht-B1a*
G153	Scorsonera	n.a.	Italy	LR	*Vrn-A1c*	*Ppd-A1b*	*Ppd-B1a*	*Rht-B1a*
G154	Cappelli	1915	Italy	OC	*Vrn-A1b*	*Ppd-A1b*	*Ppd-B1b*	*Rht-B1a*
G155	Cappelli	1915	Italy	OC	*Vrn-A1b*	*Ppd-A1b*	*Ppd-B1a*	*Rht-B1a*
G156	Simeto	1988	Italy	MC	*Vrn-A1c*	*Ppd-A1b*	*Ppd-B1a*	*Rht-B1b*
G157	Soldur	n.a.	n.a.	MC	*Vrn-A1*	*Ppd-A1a(GS105)*	*Ppd-B1a*	*Rht-B1b*
G158	Svevo	1996	Italy	MC	*Vrn-A1c*	*Ppd-A1a(GS105)*	*Ppd-B1b*	*Rht-B1b*

G159	Svevo_Soft	2020	Italy	MC	*Vrn-A1c*	*Ppd-A1a(GS105)*	*Ppd-B1b*	*Rht-B1b*
G160	SY_Cysco	n.a.	Italy	MC	*Vrn-A1c*	*Ppd-A1b*	*Ppd-B1a*	*Rht-B1b*
G161	SY_Gilbratar	2011	Italy	MC	*Vrn-A1c*	*Ppd-A1a(GS105)*	*Ppd-B1b*	*Rht-B1b*
G162	Timilia_rb	n.a.	Italy	OC	*Vrn-A1c*	*Ppd-A1b*	*Ppd-B1a*	*Rht-B1a*
G163	Timilia3_14	n.a.	Italy	LR	*Vrn-A1c*	*Ppd-A1b*	*Ppd-B1a*	*Rht-B1a*
G164	Timilia5_25	n.a.	Italy	LR	*Vrn-A1c*	*Ppd-A1b*	*Ppd-B1a*	*Rht-B1a*
G165	Timilia7_13	n.a.	Italy	LR	*Vrn-A1c*	*Ppd-A1b*	*Ppd-B1b*	*Rht-B1a*
G166	Timilia9_15	n.a.	Italy	LR	*Vrn-A1*	*Ppd-A1b*	*Ppd-B1a*	*Rht-B1a*
G167	Timilia	n.a.	Italy	LR	*Vrn-A1c*	*Ppd-A1b*	*Ppd-B1a*	*Rht-B1a*
G168	Tirex	2007	Italy	MC	*Vrn-A1c*	*Ppd-A1a(GS105)*	*Ppd-B1b*	*Rht-B1b*
G169	Tito_Flavio	1976	Italy	MC	*Vrn-A1c*	*Ppd-A1b*	*Ppd-B1a*	*Rht-B1b*
G170	Tito	2013	Italy	MC	*Vrn-A1c*	*Ppd-A1b*	*Ppd-B1a*	*Rht-B1a*
G171	Tiziana	2001	Italy	MC	*Vrn-A1c*	*Ppd-A1b*	*Ppd-B1a*	*Rht-B1b*
G172	Trigu_Cano	n.a.	Italy	LR	*Vrn-A1b*	*Ppd-A1b*	*Ppd-B1b*	*Rht-B1a*
G173	Trinakria	1970	Italy	OC	*Vrn-A1c*	*Ppd-A1b*	*Ppd-B1a*	*Rht-B1a*
G174	Tripolino	n.a.	Italy	OC	*Vrn-A1c*	*Ppd-A1b*	*Ppd-B1b*	*Rht-B1a*
G175	UC1113	n.a.	USA	MC	*Vrn-A1c*	*Ppd-A1b*	*Ppd-B1a*	*Rht-B1a*
G176	Valbelice	1992	Italy	MC	*Vrn-A1c*	*Ppd-A1b*	*Ppd-B1a*	*Rht-B1b*
G177	Valforte	1980	Italy	MC	*Vrn-A1c*	*Ppd-A1b*	*Ppd-B1a*	*Rht-B1b*
G178	Valgerardo	n.a.	Italy	MC	*Vrn-A1c*	*Ppd-A1b*	*Ppd-B1a*	*Rht-B1b*
G179	Valnova	1975	Italy	MC	*Vrn-A1c*	*Ppd-A1b*	*Ppd-B1a*	*Rht-B1b*
G180	Valriccardo	1980	Italy	MC	*Vrn-A1b*	*Ppd-A1b*	*Ppd-B1a*	*Rht-B1b*
G181	Vera_63	1964	Italy	OC	*Vrn-A1*	*Ppd-A1b*	*Ppd-B1a*	*Rht-B1a*
G182	Vitromax	1996	Italy/Spain	MC	*Vrn-A1c*	*Ppd-A1b*	*Ppd-B1a*	*Rht-B1b*
G183	Windur	2014	n.a.	MC	*Vrn-A1b*	*Ppd-A1b*	*Ppd-B1a*	*Rht-B1b*
G184	Wintergold	2014	n.a.	MC	*Vrn-A1c*	*Ppd-A1a(GS105)*	*Ppd-B1a*	*Rht-B1b*
G185	Yukon	2014	n.a.	MC	*Vrn-A1*	*Ppd-A1b*	*Ppd-B1a*	*Rht-B1b*
G186	Zingariello	n.a.	Italy	LR	*Vrn-A1c*	*Ppd-A1b*	*Ppd-B1a*	*Rht-B1a*

Supplementary Table 2. Summary statistics for five yield-related traits in durum wheat evaluated across multiple environments from 2021 to 2024. For each yield-related traits Minimum (Min), Maximum (Max) values, together with the Standard Deviation (SD), Coefficient of Variation, and broad-sense heritability (h^2) were shown across all sowing-season combinations.

Trait	Sowing-season combination	Min	Max	SD	Coefficient of Variation	h^2
GN	1_2021-2022	21.250	83.650	12.525	0.247	
GN	2_2021-2022	23.833	78.500	10.865	0.211	
GN	3_2021-2022	15.400	72.667	9.441	0.201	
GN	1_2022-2023	24.118	65.802	8.207	0.171	0.502
GN	2_2022-2023	16.861	60.477	8.055	0.197	0.537
GN	3_2022-2023	20.749	44.085	4.953	0.154	0.352
GN	2_2023-2024	9.890	49.081	7.270	0.251	0.553
GN	3_2023-2024	12.105	46.291	5.101	0.172	0.438
GW	1_2021-2022	0.483	3.518	0.562	0.282	
GW	2_2021-2022	0.588	3.447	0.559	0.264	
GW	3_2021-2022	0.565	2.757	0.443	0.243	
GW	1_2022-2023	0.756	2.277	0.336	0.223	0.484
GW	2_2022-2023	0.453	1.897	0.274	0.268	0.533
GW	3_2022-2023	0.343	0.963	0.105	0.200	0.315
GW	2_2023-2024	0.827	1.681	0.156	0.132	0.302
GW	3_2023-2024	0.420	1.658	0.208	0.197	0.395
NS	1_2021-2022	11.350	28.100	2.239	0.102	
NS	2_2021-2022	14.800	28.333	1.962	0.090	
NS	3_2021-2022	16.333	25.667	2.071	0.099	
NS	1_2022-2023	18.423	27.983	1.582	0.070	0.502
NS	2_2022-2023	17.282	26.278	1.837	0.085	0.642
NS	3_2022-2023	15.640	22.921	1.366	0.072	0.586
NS	2_2023-2024	11.907	24.117	2.171	0.113	0.524
NS	3_2023-2024	14.432	20.927	1.209	0.070	0.413
SL	1_2021-2022	5.075	12.360	1.234	0.147	
SL	2_2021-2022	5.583	12.417	1.291	0.156	
SL	3_2021-2022	5.750	12.083	1.378	0.162	
SL	1_2022-2023	6.702	14.555	1.135	0.127	0.674
SL	2_2022-2023	6.236	12.289	1.133	0.134	0.736
SL	3_2022-2023	5.919	10.921	1.012	0.128	0.727
SL	2_2023-2024	5.993	12.330	1.195	0.149	0.722
SL	3_2023-2024	4.765	9.656	0.892	0.133	0.682
SW	1_2021-2022	0.753	4.770	0.691	0.228	
SW	2_2021-2022	0.913	4.590	0.708	0.236	
SW	3_2021-2022	1.245	4.880	0.610	0.214	
SW	1_2022-2023	1.221	3.270	0.414	0.172	0.482
SW	2_2022-2023	1.031	2.931	0.343	0.189	0.540
SW	3_2022-2023	0.692	2.062	0.209	0.171	0.447
SW	2_2023-2024	1.252	2.418	0.224	0.128	0.355
SW	3_2023-2024	0.669	2.509	0.316	0.196	0.495

Abbreviations: GN = *grain number per spike;* GW = *grain weight per spike*; NS = *number of spikelets per spike*; SL= *spike length*; SW = *spike weight*; HD = *heading date*; PH = *plant height;* "1_" = *early sowing date;* "2_" = *optimal sowing date;* "3_" = *delayed sowing date.*

**Supplementary Table 3.** Top-20 Genomic prediction (GP) models used to predict Grain Number per spike (GN).			
**GP model**	**Sowing-by-season combination**	**Prediction ability (r)**	**Prediction ability (sd)**
MTME_CV2_G	2_2021-2022	0.8402	0.0446
MT_CV2_G	2_2023-2024	0.8335	0.0465
MTME_CV2_G	1_2022-2023	0.8329	0.0580
MTME_CV2_G	1_2021-2022	0.8238	0.0429
MTME_CV2_G	3_2021-2022	0.8235	0.0528
MTME_CV2_G	2_2022-2023	0.8123	0.0650
MTME_CV2_G	2_2023-2024	0.8085	0.0684
MT_CV2_G	2_2021-2022	0.7653	0.0633
MT_CV2_G	1_2022-2023	0.7361	0.0957
MT_CV2_G	1_2021-2022	0.7285	0.0716
MT_CV2_G	2_2022-2023	0.7135	0.1139
MTME_CV2_G	3_2022-2023	0.7047	0.0947
MT_CV2_G	3_2021-2022	0.7012	0.0822
MT_CV2_G	3_2022-2023	0.6741	0.0903
ME_CV2_G2	2_2021-2022	0.6273	0.0890
ME_CV2_G2	2_2023-2024	0.6217	0.0894
MT_CV1_G2	2_2023-2024	0.6207	0.0743
MT_CV2_G2	2_2023-2024	0.6207	0.0743
ME_CV1_G2	2_2023-2024	0.6189	0.0893
SE_G2	2_2023-2024	0.6139	0.0913

Abbreviations: G = *Genomic relationship matrix*; G2 = *Target gene-based relationship matrix*; SE = *Single-trait and Single-environment*; MT = *Multi-trait and Single-environment*; ME = *Single-trait and Multi-environment*; MTME = *Multi-trait and Multi-environment*; CV = *Cross-Validation;* "1_" = *early sowing date;* "2_" = *optimal sowing date;* "3_" = *delayed sowing date.*

**Supplementary Table 4.** Top-20 GP models used to predict Grain Weight per spike (GW).			
**GP model**	**Sowing-by-season combination**	**Prediction ability (r)**	**Prediction ability (sd)**
MT_CV2_G	2_2023-2024	0.8659	0.0464
MT_CV2_G	1_2022-2023	0.8311	0.0557
MTME_CV2	2_2021-2022	0.8209	0.0493
MTME_CV2	3_2021-2022	0.8065	0.0640
MT_CV2_G	2_2022-2023	0.8042	0.0523
MTME_CV2	1_2022-2023	0.7967	0.0643
MTME_CV2	1_2021-2022	0.7962	0.0635
MTME_CV2	2_2022-2023	0.7933	0.0733
MT_CV2_G	2_2021-2022	0.7916	0.0664
MT_CV2_G	3_2021-2022	0.7761	0.0692
MT_CV2_G	1_2021-2022	0.7506	0.0606
MTME_CV2	2_2023-2024	0.7458	0.0787
MT_CV2_G	3_2023-2024	0.7456	0.0649
MT_CV2_G	3_2022-2023	0.7403	0.0597
MTME_CV2	3_2023-2024	0.7024	0.0686
MTME_CV2	3_2022-2023	0.5874	0.0982
MT_CV1_G	2_2023-2024	0.5857	0.0965
ME_CV1_G2	2_2023-2024	0.5855	0.0944
SE_G	2_2023-2024	0.5843	0.0993
SE_G2	2_2023-2024	0.5834	0.1017

Abbreviations: G = *Genomic relationship matrix*; G2 = *Target gene-based relationship matrix*; SE = *Single-trait and Single-environment*; MT = *Multi-trait and Single-environment*; ME = *Single-trait and Multi-environment*; MTME = *Multi-trait and Multi-environment*; CV = *Cross-Validation*; "1_" = *early sowing date;* "2_" = *optimal sowing date;* "3_" = *delayed sowing date.*

**Supplementary Table 5.** Top-20 GP models used to predict Number of Spikelets per spike (NS).			
**GP model**	**Sowing-by-season combination**	**Prediction ability (r)**	**Prediction ability (sd)**
MTME_CV2_G	3_2021-2022	0.8685	0.0354
MTME_CV2_G	2_2022-2023	0.8498	0.0453
MTME_CV2_G	3_2022-2023	0.8150	0.0556
MTME_CV2_G	2_2021-2022	0.8092	0.0636
MTME_CV2_G	1_2021-2022	0.8069	0.0703
MTME_CV2_G	1_2022-2023	0.7783	0.0748
MT_CV2_G	3_2021-2022	0.7562	0.0897
MTME_CV2_G	3_2023-2024	0.7188	0.0675
ME_CV2_G2	3_2021-2022	0.7169	0.0672
ME_CV2_G	3_2021-2022	0.7075	0.0097
MTME_CV2_G	2_2023-2024	0.6996	0.0950
MT_CV1_G2	3_2021-2022	0.6847	0.0788
MT_CV2_G2	3_2021-2022	0.6847	0.0788
ME_CV2_G2	2_2022-2023	0.6771	0.0750
MTME_CV1_G2	3_2021-2022	0.6767	0.0873
MTME_CV2_G2	3_2021-2022	0.6767	0.0873
SE_G2_G	3_2021-2022	0.6718	0.0770
ME_CV1_G2	3_2021-2022	0.6646	0.0922
ME_CV2_G	2_2022-2023	0.6613	0.0116
ME_CV2_G	3_2022-2023	0.6542	0.0110

Abbreviations: G = *Genomic relationship matrix*; G2 = *Target gene-based relationship matrix*; SE = *Single-trait and Single-environment*; MT = *Multi-trait and Single-environment*; ME = *Single-trait and Multi-environment*; MTME = *Multi-trait and Multi-environment*; CV = *Cross-Validation*; "1_" = *early sowing date;* "2_" = *optimal sowing date;* "3_" = *delayed sowing date.*

Supplementary Table 6. Top-20 GP models used to predict Spike Length (SL).

GP model	Sowing-by-season combination	Prediction ability (r)	Prediction ability (sd)
MTME_CV2_G	2_2021-2022	0.9053	0.0347
MTME_CV2_G	3_2021-2022	0.8978	0.0471
MTME_CV2_G	3_2022-2023	0.8896	0.0365
MTME_CV2_G	1_2021-2022	0.8837	0.0353
MTME_CV2_G	3_2023-2024	0.8423	0.0527
MTME_CV2_G	1_2022-2023	0.8270	0.0784
ME_CV2_G2	2_2022-2023	0.8224	0.0628
ME_CV2_G	2_2022-2023	0.8183	0.0091
ME_CV2_G2	2_2021-2022	0.8117	0.0666
ME_CV2_G	3_2022-2023	0.8037	0.0067
ME_CV2_G2	3_2022-2023	0.7996	0.0473
ME_CV2_G	2_2021-2022	0.7990	0.0092
MTME_CV2	2_2023-2024	0.7947	0.0726
ME_CV2_G2	3_2021-2022	0.7829	0.0869
ME_CV2_G	1_2021-2022	0.7803	0.0090
ME_CV2_G2	1_2021-2022	0.7793	0.0637
ME_CV2_G	3_2021-2022	0.7666	0.0112
MT_CV2_G	3_2021-2022	0.7345	0.0636
ME_CV2_G2	3_2023-2024	0.7311	0.0860
SE_G2	2_2023-2024	0.7204	0.0917

Abbreviations: G = *Genomic relationship matrix*; G2 = *Target gene-based relationship matrix*; SE = *Single-trait and Single-environment*; MT = *Multi-trait and Single-environment*; ME = *Single-trait and Multi-environment*; MTME = *Multi-trait and Multi-environment*; CV = *Cross-Validation*; "1_" = *early sowing date;* "2_" = *optimal sowing date;* "3_" = *delayed sowing date.*

Supplementary Table 7. Top-20 GP models used to predict Spike Weight (SW).			
**GP model**	**Sowing-by-season combination**	**Prediction ability (r)**	**Prediction ability (sd)**
MT_CV2_G	2_2023-2024	0.8457	0.0387
MTME_CV2_G	2_2021-2022	0.8171	0.0507
MT_CV2_G	2_2021-2022	0.8068	0.0524
MTME_CV2_G	1_2021-2022	0.7980	0.0695
MT_CV2_G	1_2022-2023	0.7975	0.0868
MTME_CV2_G	3_2021-2022	0.7889	0.0667
MT_CV2_G	2_2022-2023	0.7802	0.0643
MTME_CV2_G	1_2022-2023	0.7582	0.0802
MTME_CV2_G	2_2022-2023	0.7571	0.0722
MT_CV2_G	3_2023-2024	0.7567	0.0624
MT_CV2_G	1_2021-2022	0.7536	0.0762
MT_CV2_G	3_2021-2022	0.7358	0.0638
MTME_CV2_G	2_2023-2024	0.7252	0.0805
MTME_CV2_G	3_2023-2024	0.7182	0.0629
MT_CV2_G	3_2022-2023	0.7058	0.0818
MTME_CV2_G	3_2022-2023	0.5820	0.1057
SE_G	2_2023-2024	0.5544	0.1082
SE_G2	2_2023-2024	0.5525	0.1112
ME_CV1_G2	2_2023-2024	0.5515	0.0922
MT_CV1_G	2_2023-2024	0.5448	0.0975

Abbreviations: G = *Genomic relationship matrix*; G2 = *Target gene-based relationship matrix*; SE = *Single-trait and Single-environment*; MT = *Multi-trait and Single-environment*; ME = *Single-trait and Multi-environment*; MTME = *Multi-trait and Multi-environment*; CV = *Cross-Validation*; “1_” = *early sowing date;* “2_” = *optimal sowing date;* “3_” = *delayed sowing date.*

Supplementary Table 8. Top-20 GP models used to predict Heading Date (HD).

**GP model**	**Sowing-by-season combination**	**Prediction ability (r)**	**Prediction ability (sd)**
MTME_CV2_G	2_2022-2023	0.9669	0.0102
MTME_CV2_G	2_2023-2024	0.9654	0.0119
MTME_CV2_G	3_2022-2023	0.9594	0.0134
MTME_CV2_G	1_2022-2023	0.9586	0.0149
MTME_CV2_G	3_2023-2024	0.9543	0.0152
MTME_CV2_G	1_2021-2022	0.9464	0.0179
MTME_CV2_G	2_2021-2022	0.9459	0.0163
ME_CV2_G2	2_2023-2024	0.9370	0.0191
ME_CV2_G2	2_2022-2023	0.9368	0.0171
ME_CV2_G2	1_2022-2023	0.9204	0.0236
ME_CV2_G2	3_2022-2023	0.9199	0.0258
MTME_CV2_G	3_2021-2022	0.9195	0.0350
ME_CV2_G2	2_2021-2022	0.9059	0.0287
ME_CV2_G2	1_2021-2022	0.9015	0.0309
ME_CV2_G2	3_2023-2024	0.8965	0.0275
ME_CV2_G2	3_2021-2022	0.8359	0.0584
MT_CV2_G	2_2023-2024	0.8185	0.0543
SE_G2	2_2023-2024	0.8141	0.0667
MT_CV1_G2	2_2023-2024	0.8128	0.0400
MT_CV2_G2	2_2023-2024	0.8128	0.0400

Abbreviations: G = *Genomic relationship matrix*; G2 = *Target gene-based relationship matrix*; SE = *Single-trait and Single-environment*; MT = *Multi-trait and Single-environment*; ME = *Single-trait and Multi-environment*; MTME = *Multi-trait and Multi-environment*; CV = *Cross-Validation*; "1_" = *early sowing date;* "2_" = *optimal sowing date;* "3_" = *delayed sowing date.*

**Supplementary Table 9.** Top-20 GP models used to predict Plant Height (PH).			
**GP model**	**Sowing-by-season combination**	**Prediction ability (r)**	**Prediction ability (sd)**
MTME_CV2_G	2_2022-2023	0.9723	0.0181
MTME_CV2_G	2_2021-2022	0.9698	0.0170
MTME_CV2_G	1_2022-2023	0.9688	0.0189
MTME_CV2_G	1_2021-2022	0.9657	0.0214
MTME_CV2_G	3_2022-2023	0.9629	0.0178
ME_CV2_G2	2_2022-2023	0.9486	0.0124
ME_CV2_G2	2_2021-2022	0.9437	0.0134
ME_CV2_G2	1_2022-2023	0.9429	0.0153
ME_CV2_G2	1_2021-2022	0.9281	0.0227
MTME_CV2_G	3_2021-2022	0.9229	0.0682
MTME_CV2_G	3_2023-2024	0.9177	0.0456
ME_CV2_G2	3_2022-2023	0.9149	0.0203
SE_G2	2_2021-2022	0.9084	0.0240
MT_CV1_G2	2_2021-2022	0.9043	0.0291
MT_CV2_G2	2_2021-2022	0.9043	0.0291
MT_CV1_G2	2_2022-2023	0.8947	0.0324
MT_CV2_G2	2_2022-2023	0.8947	0.0324
SE_G2	2_2022-2023	0.8944	0.0336
ME_CV1_G2	2_2021-2022	0.8920	0.0279
MTME_CV1_G2	2_2021-2022	0.8914	0.0283
Abbreviations: G = *Genomic relationship matrix*; G2 = *Target gene-based relationship matrix*; SE = *Single-trait and Single-environment*; MT = *Multi-trait and Single-environment*; ME = *Single-trait and Multi-environment*; MTME = *Multi-trait and Multi-environment*; CV = *Cross-Validation*; “1_” = *early sowing date;* “2_” = *optimal sowing date;* “3_” = *delayed sowing date.*			
