# Supplementary material for: Multi-trait and multi-environment genomic prediction enhances yield components improvement in durum wheat
Source: Front Plant Sci. 2026 Feb 23;17:1759897. doi: 10.3389/fpls.2026.1759897 (PMC12968193; doi:10.3389/fpls.2026.1759897)
Supplement: Supplementary file 2 [file DataSheet2.pdf]

frontiers in Plant Science

Multi-trait and Multi-environment Genomic Prediction Enhances Yield Components Improvement in Durum Wheat

D. Puglisi[1], J. Crossa[2], J. Cuevas[3], F. Fania[1], P. Vitale[4], and P. De Vita[1]

[1]CREA – Research Centre for Cereal and Industrial Crops (CREA-CI) Consiglio per la Ricerca in Agricoltura e l'Analisi dell'Economia Agraria S.S. 673, m 25,200 71122 Foggia (FG), Italy
[2]Colegio de Postgraduados, Campus Montecillo, Carretera México-Texcoco, Km. 36.5, Montecillo, Texcoco, Estado de México C.P. 56264, México
[3]Division de Ciencias, Ingeniería y Tecnología (DCIT), Universidad Autónoma del Estado de Quintana Roo, Chetumal, Quintana Roo, 77019, México
[4]International Maize and Wheat Improvement Center (CIMMYT) Carretera México-Veracruz, Km. 45, El Batán, Texcoco, Estado de México 56237 México

Supplementary Figures

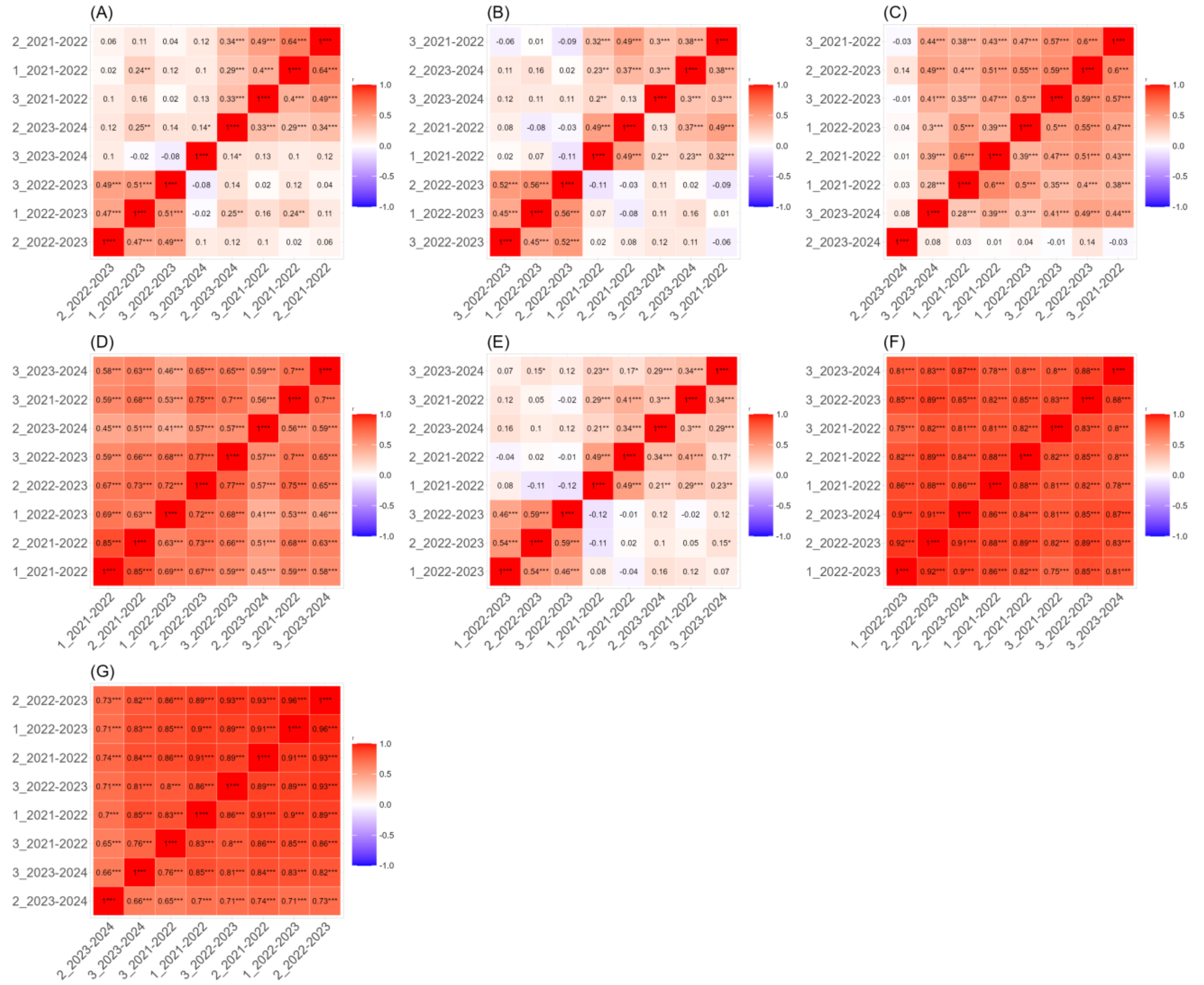

Supplementary Figure 1. Heatmap of Pairwise Pearson's correlations across sowing-by-season combination for each yield-related and morpho-phenological traits: (A) Grain Number per spike (GN), **(B)** Grain Weight per spike (GW), **(C)** Number of Spikelets per spike (NS), **(D)** Spike Length (SL), **(E)** Spike Weight (SW), **(F)** Heading Date (HD), and **(G)** Plant Height (PH). The asterisks indicate the significance of the correlation (***<0.001, **<0.01, *<0.05).

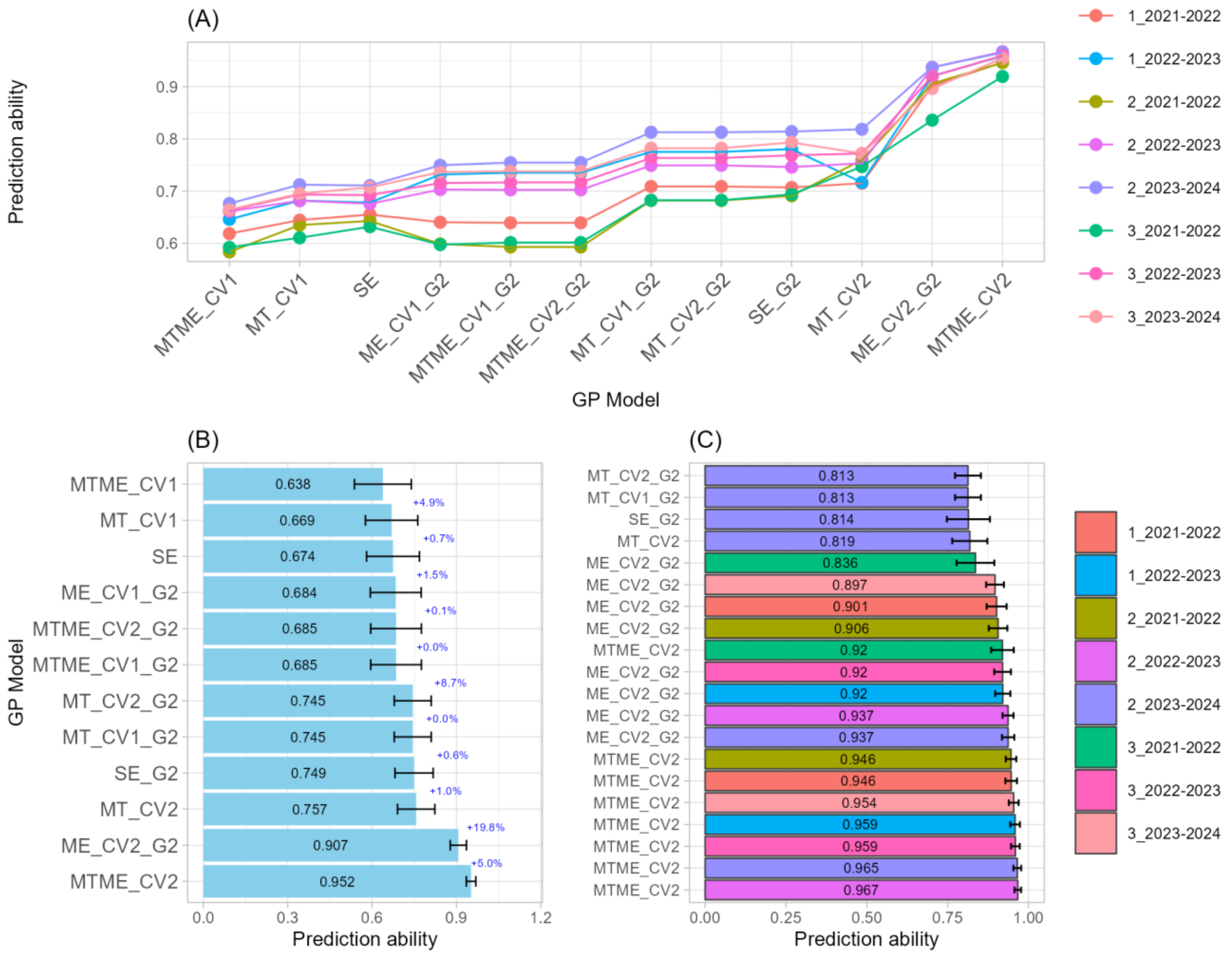

Supplementary Figure 2. Single-trait-single-environment (SE), multi-trait-single-environment (MT), single-trait-multi-environment (ME), and multi-trait–multi-environment (MTME) genomic prediction (GP) models used to predict Heading Date (HD) using both genomic (G) and allelic (G2) relationship matrices with two cross-validation schemes (CV1 and CV2). **(A)** Overall prediction ability for each sowing-by-season (environment) combination and GP models used; **(B)** Prediction ability across sowing-by-season (environment) combination for each GP models used. Error bars indicate variability across environments, while percentage values indicate the improvement relative to the model immediately above; **(C)** Prediction ability of top-20 GP models used.

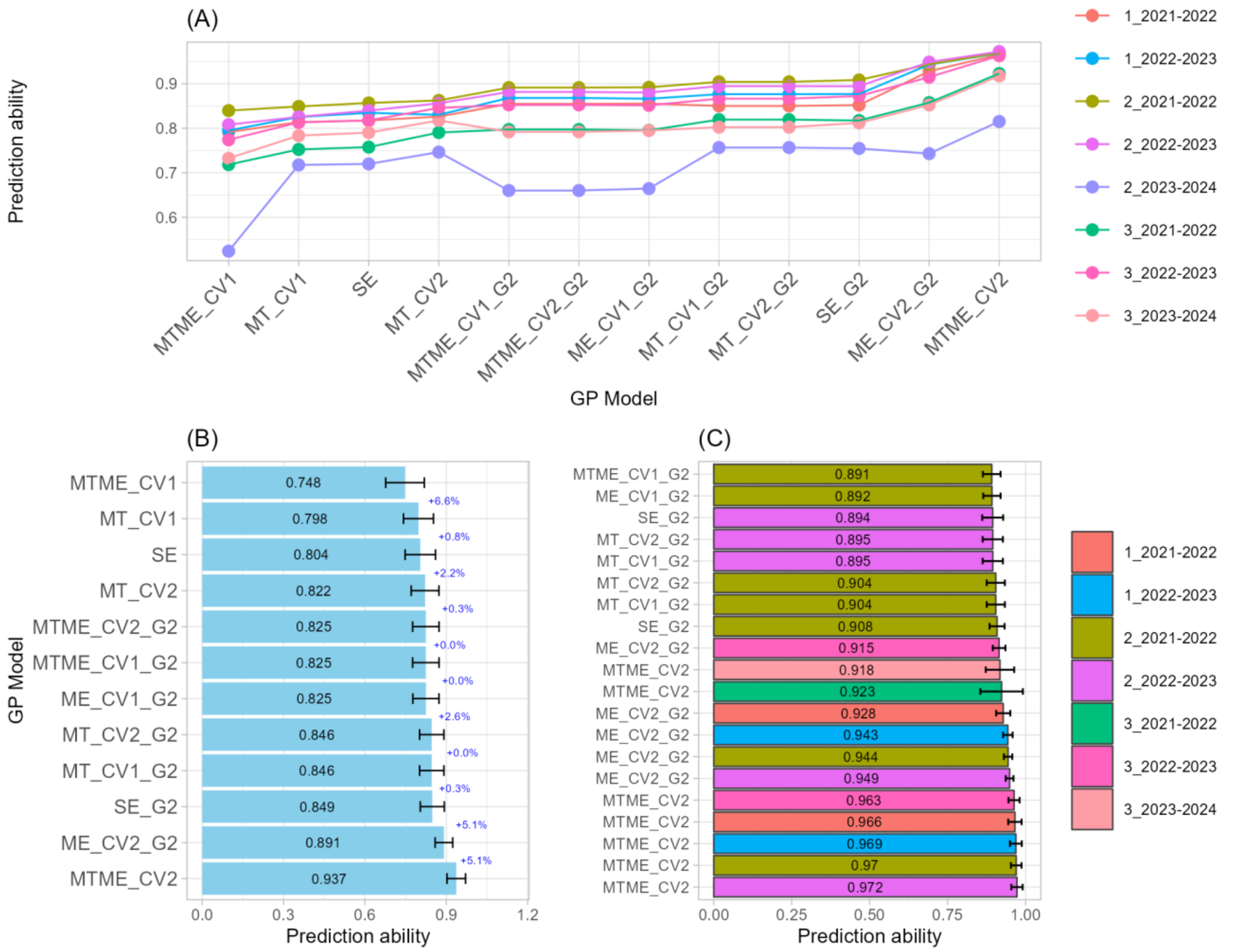

Supplementary Figure 3. Single-trait-single-environment (SE), multi-trait-single-environment (MT), single-trait-multi-environment (ME), and multi-trait–multi-environment (MTME) genomic prediction (GP) models used to predict Plant Height (PH) using both genomic (G) and allelic (G2) relationship matrices with two cross-validation schemes (CV1 and CV2). **(A)** Overall prediction ability for each sowing-by-season (environment) combination and GP models used; **(B)** Prediction ability across sowing-by-season (environment) combination for each GP models used. Error bars indicate variability across environments, while percentage values indicate the improvement relative to the model immediately above; **(C)** Prediction ability of top-20 GP models used.
